# Identification and mechanism prediction of mulberroside A metabolites *in vivo* and *in vitro* of rats using an integrated strategy of UHPLC-Q-Exactive Plus Orbitrap MS and network pharmacology

**DOI:** 10.3389/fchem.2022.981173

**Published:** 2022-09-27

**Authors:** Xiao Zhang, Pingping Dong, Jian Song, Huimin Zhang, Feiran Wang, Yuecheng Liu, Yingying Yan, Linlin Li

**Affiliations:** ^1^ Shandong University of Traditional Chinese Medicine, Jinan, China; ^2^ Shandong Academy of Chinese Medicine, Jinan, China; ^3^ State Key Laboratory for Quality Research of Chinese Medicines, Macau University of Science and Technology, Taipa, Macao SAR, China

**Keywords:** mulberroside A, UHPLC-Q-Exactive Plus Orbitrap MS, network pharmacology, metabolites, metabolic pathways

## Abstract

Mulberroside A is a polyhydroxylated stilbene active component of *Morus alba* L. Studies have shown that it has antitussive, antiasthmatic, tyrosinase and antioxidation activities. However, little is known about the metabolism of it *in vitro* and *in vivo*. In our study, an integrated strategy on the basis of UHPLC-Q-Exactive Plus Orbitrap MS and network pharmacology was established to comprehensively research the metabolic characteristic of mulberroside A for the first time. Plasma, urine, feces and liver tissues of rats in the blank group and drug group were collected after intragastric administration of mulberroside A at a dose of 150 mg/kg, and rat liver microsomes were cultured for *in vitro* metabolism experiment. The biological samples were processed by different methods and analyzed in positive and negative ion modes using UHPLC-Q-Exactive Plus Orbitrap MS. A total of 72 metabolites were finally identified based on the accurate molecular mass, retention time, MS/MS spectra and related literatures combined with the Compound Discoverer 3.1. The metabolic pathways were mainly hydrolysis, glucuronidation, hydrogenation, sulfation, hydroxylation, methylation and their composite reactions. In addition, a network pharmacology method was used to predict the mechanism of action of mulberroside A and its metabolites. In the end, 7 metabolites with high gastrointestinal absorption and drug-likeness and 167 targets were screened by Swiss ADME and Swiss Target Prediction. 1702 items of GO analysis and 158 related signaling pathways of KEGG were enriched using Metascape. This study established a novel integrated strategy based on UHPLC-Q-Exactive Plus Orbitrap MS and network pharmacology, which could systematically analyze the metabolism behavior of mulberroside A *in vivo* and *in vitro* of rats and provide basis for the further research of mulberroside A.

## 1 Introduction

Mulberroside A, presenting at relatively high abundance in the roots and twigs of *Morus alba* L. (Zhou et al., 2013), is a natural polyhydroxylated stilbene compound (Yu et al., 2021). Furthermore, it possesses various therapeutic effects through pharmacological studies, including hypoglycemic (Li et al., 2014), antidiabetic (Xu et al., 2021), anti-inflammatory (Chung et al., 2003), analgesic (Zhang and Shi, 2010), hepatic protective function (Zhang et al., 2008), nephroprotective (Wang et al., 2011), the inhibitory effect of melanogenesis (Park et al., 2011) and neuroprotective (Wang C. P. et al., 2014) effects, etc.

Drug metabolism, also known as drug biotransformation, refers to the process of chemical structure change of drugs under the action of drug enzymes (Jiang et al., 2014). Once a drug enters the body, it is usually converted into various forms through different metabolic pathways (Prasad et al., 2011). Drug metabolism research can elucidate the scientific connotation of drugs, clarify the pharmacodynamic active ingredients, guide the rational use of clinical drugs, and promote the improvement of dosage forms and quality control, which has great significance in the drug discovery process. However, as far as we know, the *in vivo* and *in vitro* metabolism study of mulberroside A has been poorly understood. A pharmacokinetic study on mulberroside A revealed that its oral absolute bioavailability was poor in rats (Qiu et al., 1996b). In Qiu F’s previous metabolic research on mulberroside A, four metabolites were isolated from the urine and bile of rats after oral administration of mulberroside A (Qiu et al., 1996a). After that, they reported the identification of 3 metabolites of mulberroside A in the gastrointestinal contents and feces of rats (Zhaxi et al., 2010). However, whether mulberroside A is metabolized prior to be absorbed into the blood has not been reported so far. Therefore, it is important to carry out a comprehensive metabolism study of mulberroside A. Then, we could better understand its metabolic mechanism and promote research for the further potential therapeutic applications.

Contemporarily, ultra-high performance liquid chromatography (UHPLC) coupled with mass spectrometry (MS), especially high-resolution mass spectrometry (HRMS), has been widely used in drug metabolism analysis (Dong et al., 2021; Yuan et al., 2021). UHPLC has a powerful separation capability, which is currently one of the most effective tools for the separation of complex components. In recent years, network pharmacology has been widely used in the field of traditional Chinese medicine with its unique advantages. The organic combination of metabolite identification and network pharmacology could effectively reveal the mechanism of drug action in the body. In our study, an integrated strategy on the basis of UHPLC-Q-Exactive Plus Orbitrap MS and network pharmacology was developed. This strategy was cost-effective to characterize chemical transformations and further gain comprehensive knowledge about mulberroside A.

## 2 Materials and methods

### 2.1 Chemicals and reagents

The reference standard, mulberroside A, was purchased from Chengdu Must Biotechnology Co., Ltd. (Sichuan, China), with purity greater than 98% determined using HPLC-UV analysis. Rat liver microsomes were purchased from Wuxi Xinrun Biotechnology Co., Ltd. (Jiangsu, China). HPLC grade methanol, formic acid, and acetonitrile were provided by Thermo Fisher Scientific (Fair Lawn, NJ, United States). Water was obtained from Watsons (Guangdong, China). Grace Pure SPE C_18_-Low solid-phase extraction cartridges (200 mg/3 ml, 59 μm, 70 Å) were purchased from Grace Davison Discovery Science (Deerfield, IL, United States).

### 2.2 Animals and drug administration

Six male SD rats (200 ± 10 g) were provided by Jinan Pengyue Experimental Animal Breeding Co., Ltd. (Shandong, China). The animals were housed in a controlled environment with the temperature held at 24 ± 2 °C, relative humidity held at 60±5%, and kept on a 12 h light/12 h dark regime. After 7 days of acclimation, the rats were randomly divided into drug group (n=3) and control group (n=3). Before the experiment, all rats were fasted for 12 h with free access to water. Mulberroside A was dissolved in normal saline. The rats in the drug group were given with a dose of 150 mg/kg/d orally for 3 days. A same dose of normal saline was administered to the rats in the control group.

### 2.3 Sample collection

#### 2.3.1 Plasma and liver tissue sample collection

0.5 ml of blood samples were taken from the suborbital venous plexus of rats at 0.5, 1, 1.5, 2, 4, 6, 12, and 24 h after the last administration. The blood samples collected at different times were mixed and placed in a centrifuge tube coated with sodium heparin. After that, they were centrifuged for 10 min at 3,500 rpm to separate plasma sample. Then, it was frozen in a refrigerator at -80°C. The centrifugation was performed on Centrifuge 5430R (Eppendorf, Germany).

24 h after the last administration, liver tissue was obtained and quenched in liquid nitrogen and then stored at -80°C. 1 g of liver tissue was added to 10 ml of normal saline to homogenize at 4°C and then centrifuged at 3,500 rpm for 10 min to obtain the supernatant.

### 2.3.2 Urine and feces sample collection

Two groups of rats were housed in the metabolic cage. The urine and feces were collected within 24 h after the last administration. The urine samples were frozen in the refrigerator at -80°C after centrifuged at 12,000 rpm for 15 min, and the feces samples were freeze-dried and ground into powder.

### 2.3.3 Liver microsomes incubation

MgCl_2_ and liver microsomes were dissolved in PBS (pH=7.4), formulated into a solution with a MgCl_2_ concentration of 3 mM and a protein concentration of 1 mg/ml. The solution was used to dissolve mulberroside A with a final drug concentration of 0.1 mg/ml. Drug group and blank group were set up. 900 µl of the above mixture was added to each well of the 6-well plate in the drug group while the blank group was given an equal volume of drug-negative solution. After preheating at 37°C for 5 min, 100 µl NADPH (final concentration of 25 mg/ml) was added to start the reaction and continue to incubate at 37°C. At 5, 10, 15, 30, 45, 60, 120, 240 min after the start of the reaction, 100 µl of the system solution was mixed with 200 µl of cold acetonitrile to terminate the reaction. Then, the supernatant was obtained after centrifuged at 20,000 rpm for 10 min.

### 2.4 Sample preparation

The SPE method for precipitation and concentration of protein and solid residues was applied to pretreat all the biological samples. Before loading and elution, 2 g feces was added to 10 ml pure water, sonicated for 0.5 h, and centrifuged to obtain the supernatant. Then, plasma, urine, feces, and liver tissues samples (1 ml) were added to SPE cartridges pretreated with methanol (3 ml) and deionized water (3 ml), respectively. After that, the SPE cartridges were successively washed with deionized water (3 ml) and methanol (3 ml). The methanol eluent was collected and dried under N_2_ at 4°C. The residue was then redissolved in 300 µl methanol and centrifuged for 15 min at 20,000 rpm (4°C).

In addition, plasma samples were treated with methanol (3 ml) and acetonitrile (3 ml), respectively. Then, they were centrifuged at 3,500 rpm. The obtained supernatant was dried under N_2_ at room temperature. The residue was then redissolved in 300 µl methanol and centrifuged for 15 min at 20,000 rpm (4°C).

All the biological samples from the same group were merged into a collective sample for the further instrumental analysis.

### 2.5 Instruments and analytical conditions

#### 2.5.1 UHPLC parameters

The separation was performed on Vanquish UHPLC (Thermo Scientific, Germany) with a ACQUITY UPLC BEH C_18_ column (2.1 × 100 mm, 1.7 μm). The flow rate was 0.3 ml/min and the injection volume was 5 µl. The column temperature was maintained at 35°C. The mobile phase consists of acetonitrile(A) and 0.1% formic acid-water (B). The gradient elution condition was set as: 0–5.0 min, 95% B; 5.0–10.0 min, 95%–70% B; 10.0–15.0 min, 70%–50% B; 15.0–32.0 min, 50%–10% B; 32.0–32.1 min, 10%–95% B; 32.1–35.0 min, 95% B.

### 2.5.2 HRMS parameters

The MS analysis was performed on Q-Exactive Plus Orbitrap MS (Thermo Scientific, Germany) with a heated electrospray ionization (HESI) source. A high-resolution mass spectrum was acquired at full scan in a mass range of *m/z* 80–*m/z* 1,200 at a resolution of 70,000 with AGC target at 3e^6^. The dd-MS^2^ data were obtained at a resolution of 17,500 with AGC target at 1e^6^. Mass spectrometric detection was performed in positive and negative ion modes. The ion source parameters were set as follows: sheath gas and auxiliary gas of nitrogen (purity ≥99.99%) with the flow rate of 45 arb and 10 arb; capillary temperature of 320 °C; spray voltage of 3,800/3,500 V (+/-); probe heater temperature of 320°C. The stepped normalized collision energy (NCE) was set at 15, 30 and 45. S-Lens RF Level was 50.00.

### 2.6 Data processing and statistical analyses

Thermo Scientific Xcalibur 3.1 workstation and Compound Discoverer 3.1 software were used to process and analyze the collected mass spectrometry data. The chemical formulas attributed to the selected peaks were calculated using a formula predictor by setting the parameters as follows: C [5–40], H [5–60], O [2–30], S [0–2], N [0–3] and the ring double bond (RDB) equivalent value [3–20]. The HRMS data obtained from UHPLC-Q-Exactive Plus Orbitrap MS was imported into the Compound Discoverer, and the structure information of mulberroside A was added to Compound Discoverer at the same time. Then, Compound Discoverer gave the information of possible metabolites according to the structural characteristics of mulberroside A, HRMS information and the corresponding databases. In addition, the metabolites were selected by Swiss ADME (http://www.swissadme.ch/) and the targets were predicted using Swiss Target Prediction (http://www.swisstargetprediction.ch/). All genes were submitted to Metascape (Zhou et al., 2019) (http://metascape.org) for GO and KEGG analysis and Cytoscape 3.8.2 was used for the construction of metabolite-target network and metabolite-target-pathway network. The visual analysis of GO and KEGG enrichment results was performed using Bioinformatics online platform (http://www.bioinformatics.com.cn/).

## 3 Results and discussion

### 3.1 The establishment of an analytical strategy

**FIGURE 1:**
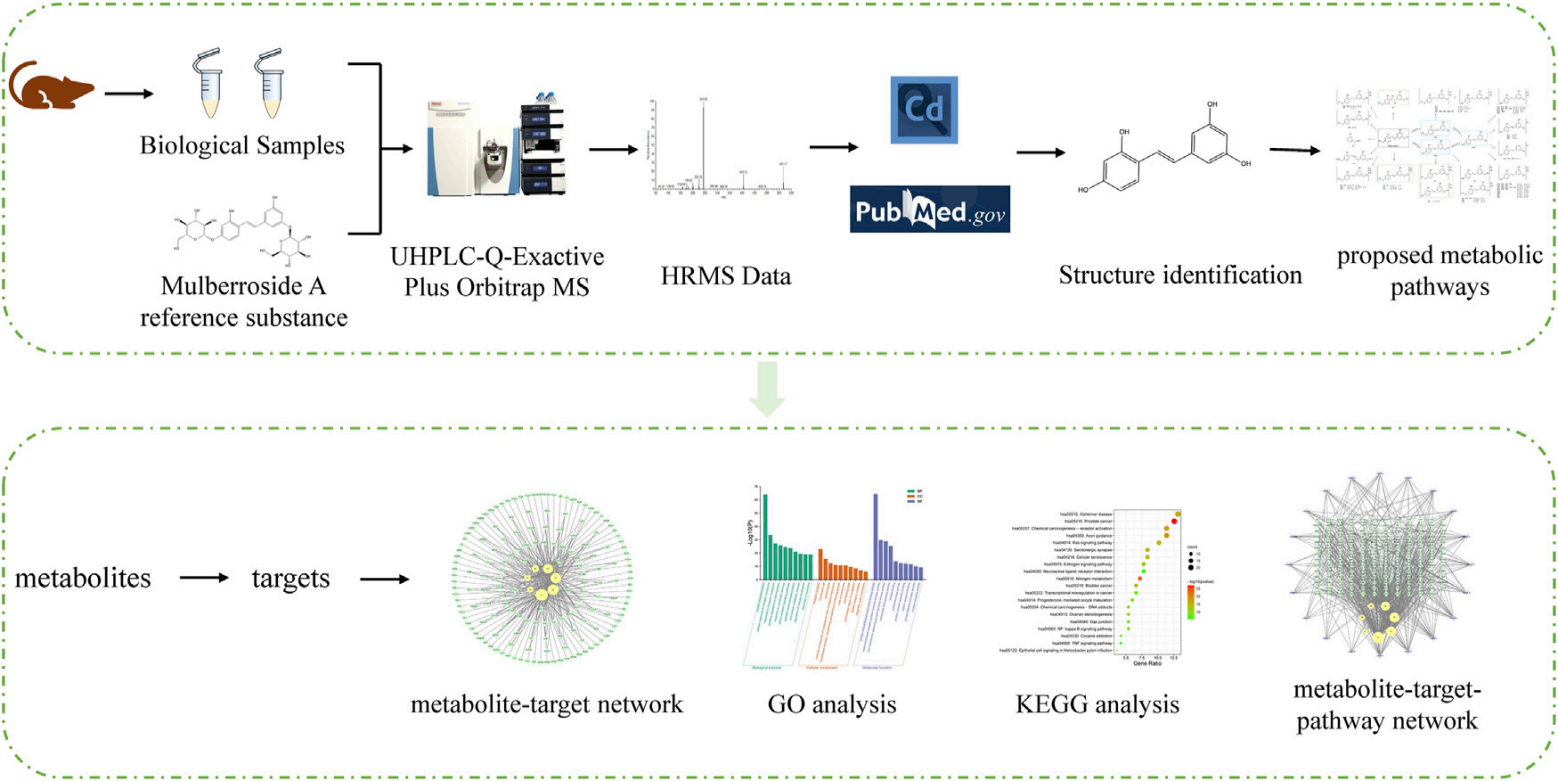
Summary diagram of the established analytical strategy.

In order to understand the metabolism and mechanism of mulberroside A comprehensively, an efficient and integrated strategy was established based on the UHPLC-Q-Exactive Plus Orbitrap MS and network pharmacology, as shown in Figure 1. (1) The rats were given the mulberroside A solution by intragastric administration and biological samples were collected. Then the treated biological samples and standard mulberroside A samples were injected into UHPLC-Q-Exactive Plus Orbitrap MS to obtain HRMS data. (2) The information such as accurate molecular mass, retention time and MS/MS spectra of compounds was obtained by Thermo Scientific Xcalibur 3.1 and then the compounds were identified based on the information and related literatures. After that, the metabolic pathway of mulberroside A could be predicted on the basis of metabolites and metabolic reactions. (3) Swiss ADME and Swiss Target Prediction were applied to screen the metabolites with high gastrointestinal absorption and drug-likeness and predict the targets of these selected metabolites, which were used to map the metabolite-target network by Cytoscape 3.8.2. Then, Metascape was used for GO and KEGG analysis. In the end, the metabolite-target-pathway network was also constructed by Cytoscape 3.8.2.

### 3.2 Analysis of the mass spectrometry fragmentation behaviors of mulberroside A

**FIGURE 2:**
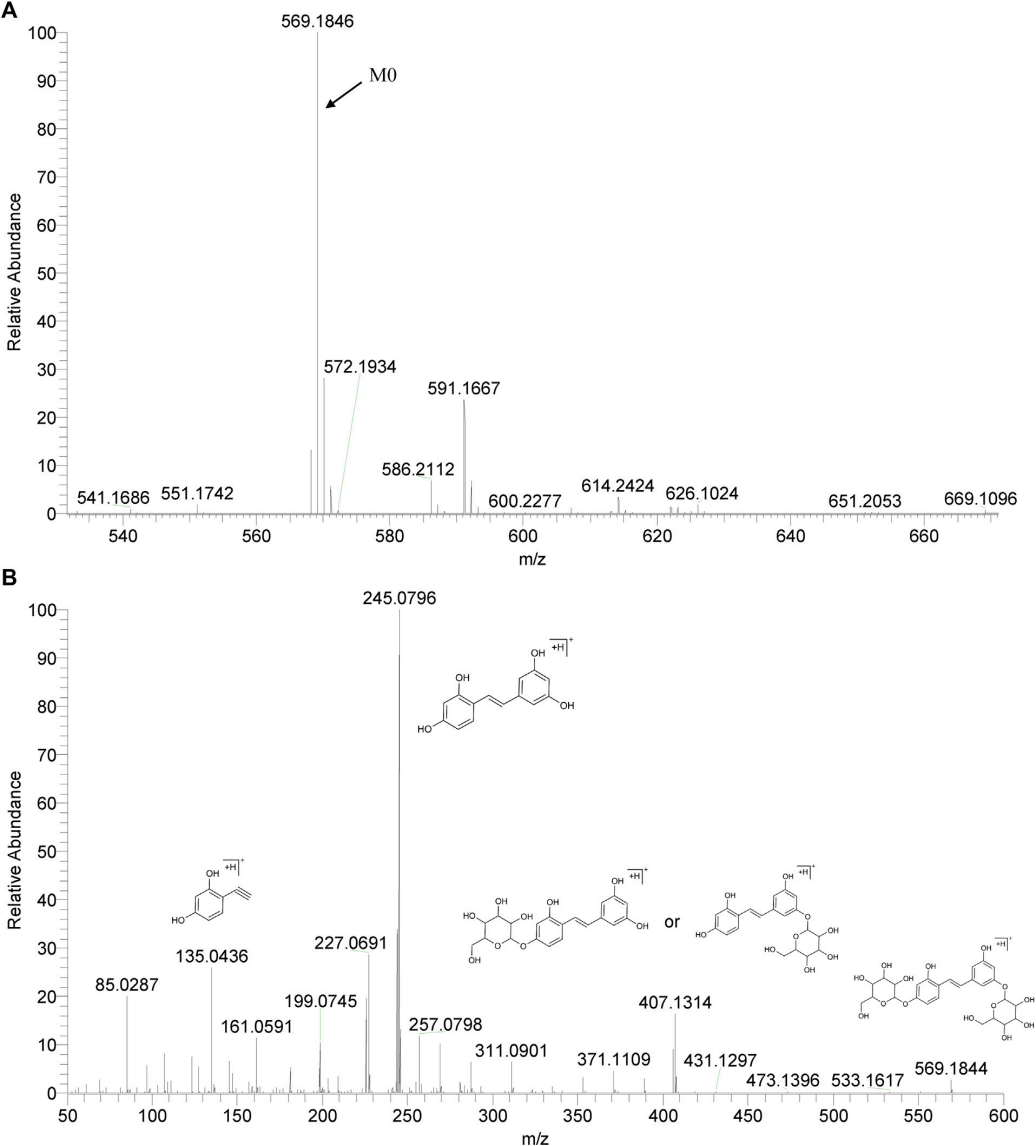
The MS **(A)** and MS2 **(B)** spectra of Mulberroside A in positive ion mode.

**FIGURE 3:**
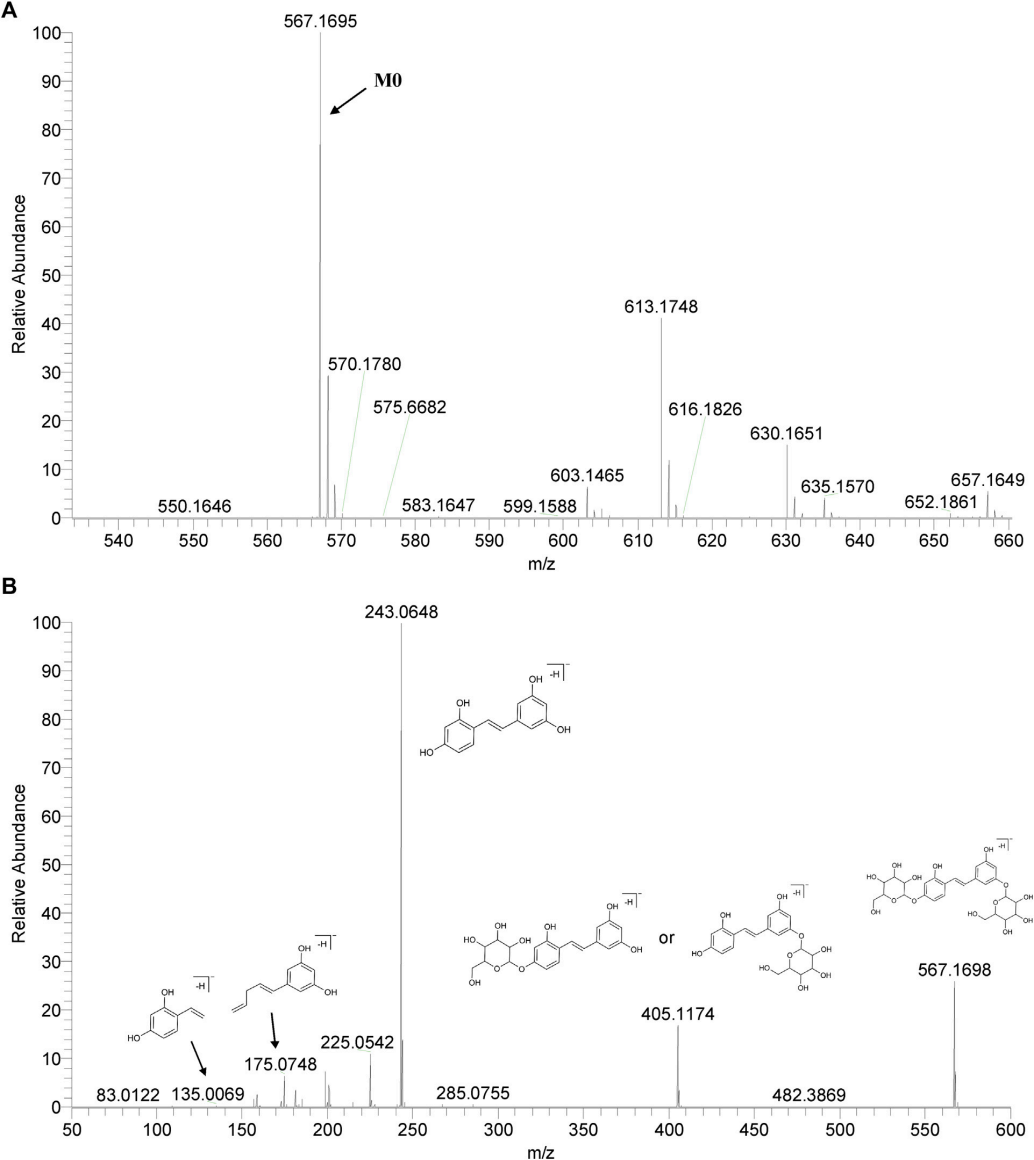
The MS **(A)** and MS2 **(B)** spectra of Mulberroside A in negative ion mode.

**FIGURE 4:**
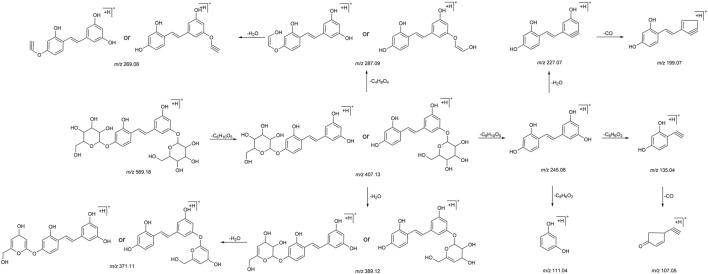
The mass fragmentation behavior of Mulberroside A in positive ion mode.

**FIGURE 5:**
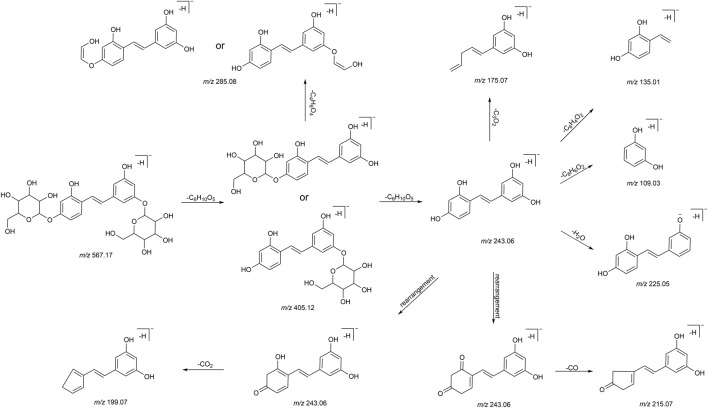
The mass fragmentation behavior of Mulberroside A in negative ion mode.

We summarized the mass spectrometry fragmentation behaviors of mulberroside A according to related literatures reported and the MS^2^ information of the reference substances (Sun, 2004; Pan et al., 2015; Xu, 2015; Guo et al., 2018; Zhang, 2018; Liang et al., 2021). As shown in Figure 2 and Figure 3, the [M+H]^+^ and [M-H]^-^ was generated in positive and negative ion modes at *m/z* 569.1846 and *m/z* 567.1695, respectively, in the ESI-MS spectra of mulberroside A. Characteristic fragments were detected in positive ion mode: *m/z* 407 [M+H-glucose]^+^ ([M+H-Glu]^+^), *m/z* 245 [M+H-2Glu]^+^, *m/z* 227 [M+H-2Glu-H_2_O]^+^, *m/z* 199 [M+H-2Glu-H_2_O-CO]^+^, *m/z* 135 [M+H-2Glu-C_6_H_6_O_2_]^+^. Representative ions were detected in negative ion mode as follows: *m/z* 405 [M-H-Glu]^-^, *m/z* 243 [M-H-2Glu]^-^, *m/z* 225 [M-H-2Glu-H_2_O]^-^, *m/z* 215 [M-H-2Glu-CO]^-^, *m/z* 199 [M-H-2Glu-CO_2_]^-^, *m/z* 181 [M-H-2Glu-CO_2_-H_2_O]^-^, *m/z* 175 [M-H-2Glu-C_3_O_2_]^−^, *m/z* 157 [M-H-2Glu-C_3_O_2_-H_2_O]^−^. The mass fragmentation behavior of mulberroside A in positive and negative ion modes are shown in Figure 4 and Figure 5 respectively.

### 3.3 Detection and structural elucidation of mulberroside A metabolites

A total of 76 metabolites were finally identified based on the accurate molecular mass, retention time, MS/MS spectra and related literature reports in the plasma, urine, feces, liver tissues and liver microsomes. The chromatographic and MS data of these detected metabolites were summarized in Table 1.

**TABLE 1:** Summary of mulberroside A metabolites *in vivo* and *in vitro* of rats.

Peak	*t*_R_/min	Ion mode	Formula **[M-H]^−^**	Theoretical Mass (*m/z*)	Experimental Mass (*m/z*)	RDB	Error (ppm)	MS/MS fragment ions	Identification/Reactions	PS	PM	PA	U	F	L	LM	Max. Peak area
M0	8.18	N	C_26_H_31_O_14_	567.1708	567.1707	11.5	−0.197	243.06(100.00), 567.17(27.20), 405.12(22.51), 225.05(10.57), 199.08(7.11), 175.07(5.26)	Mulberroside A	√	√	√				√	1.53E+06 (PM)
M1*	5.55	N	C_12_H_13_O_8_	285.0605	285.0607	6.5	0.618	109.03(100.00), 113.02(61.96), 285.06(38.68), 175.02(18.76)	β-D-Glucopyranosiduronic Acid 3-Hydroxyphenyl	√	√	√	√				2.53E+08 (U)
M2	7.72	N	C_26_H_31_O_15_	583.1652	583.1658	11.5	0.143	259.06(100.00), 583.17(45.98), 421.11(32.65), 215.07(2.51)	hydroxylation							√	2.08E+06 (LM)
M3	7.98	N	C_26_H_29_O_15_	581.1501	581.1499	12.5	-0.338	581.15(100.00), 243.06(52.49), 225.05(6.60)	hydrolysis	√	√						2.25E+06 (PS)
									glucuronidation								
M4	8.18	N	C_26_H_33_O_18_S	665.1382	665.1384	10.5	0.239	96.96(100.00), 243.07(5.60), 567.17(4.23), 665.15(3.58), 405.12(0.52)	hydrogenation				√				3.58E+07 (U)
									hydroxylation								
									sulfation								
M5	8.19	N	C_27_H_33_O_16_	613.1763	613.1750	11.5	-2.155	243.07(100.00), 567.17(43.17), 225.05(7.89), 405.12(6.46), 435.56(0.28), 448.96(0.28)	dihydroxylation	√	√	√					5.81E+05 (PS)
									methylation								
M6	8.20	N	C_26_H_31_O_15_	583.1652	583.1658	11.5	-1.314	259.06(100.00), 421.11(55.47), 583.17(39.04), 241.05(18.86)	hydroxylation							√	8.12E+06 (LM)
M7	8.20	N	C_26_H_31_O_16_	599.1607	599.1602	11.5	-0.736	137.02(100.00), 599.16(57.05), 125.02(50.33), 437.11(45.05), 275.06(25.62)	dihydroxylation							√	3.98E+06 (LM)
M8	8.20	N	C_20_H_21_O_11_	437.1078	437.1079	10.5	0.042	137.02(100.00), 437.11(72.57), 257.04(56.49), 275.05(30.75)	hydrolysis							√	3.16E+06 (LM)
									dihydroxylation								
M9	8.24	N	C_26_H_33_O_16_	601.1763	601.1769	10.5	1.046	137.02(100.00), 243.07(42.31), 601.18(33.69), 567.17(20.17), 439.12(13.78)	hydrogenation							√	2.73E+06 (LM)
									dihydroxylation								
M10	8.29	N	C_21_H_21_O_10_	433.1129	433.1125	11.5	-1.000	313.07(100.00), 433.11(84.30), 343.08(36.15), 257.08(17.32), 243.07(10.86)	hydrolysis					√			1.39E+07 (F)
									methylation								
									glucuronidation								
M11	8.43	N	C_26_H_29_O_16_	597.1450	597.1450	12.5	0.015	597.14(100.00), 421.11(99.27), 245.08(21.33), 175.02(22.04), 135.04(9.32), 243.06(7.70), 157.01(3.09)	hydrolysis				√				5.97E+07 (U)
									hydrogenation								
									bis-glucuronidation								
M12	8.52	N	C_20_H_17_O_10_	417.0816	417.0803	12.5	−3.268	417.17(100.00), 197.13(7.56), 241.12(7.42)	hydrolysis					√			3.16E+04 (F)
									dehydrogenation								
									glucuronidation								
M13	8.57	N	C_26_H_31_O_15_	583.1658	583.1650	11.5	−1.212	583.17(100.00), 245.01(27.22), 421.11(1.19)	hydrolysis				√				8.16E+06 (U)
									hydrogenation								
									glucuronidation								
M14	8.62	N	C_32_H_39_O_21_	759.1978	759.1960	13.5	−2.364	421.11(100.00), 245.08(9.61), 175.02(7.66), 135.04(3.69), 109.03(3.24), 583.47(0.27)	hydrolysis				√				8.17E+06 (U)
									hydrogenation								
									bis-glucuronidation								
M15	8.76	N	C_26_H_27_O_16_	595.1294	595.1291	13.5	−0.455	419.10(100.00), 595.13(76.29), 243.07(75.87), 175.02(18.03)	hydrolysis	√		√	√				1.02E+08 (U)
									bis-glucuronidation								
M16	8.77	N	C_26_H_29_O_15_	581.1501	581.1495	12.5	−0.958	581.15(100.00), 243.07(73.83), 405.12(3.42), 225.05(4.57)	Hydrolysis	√							2.86E+06 (PS)
									Glucuronidation								
M17	8.80	N	C_20_H_19_O_13_S	499.0541	499.0544	11.5	0.706	499.05(100.00), 243.07(85.68), 323.02(55.07), 225.05(11.54), 419.09(10.13)	Hydrolysis		√	√					1.29E+06 (PM)
									Glucuronidation								
									sulfation								
M18	8.83	N	C_20_H_21_O_13_S	501.0697	501.0696	10.5	−0.315	501.07(68.38), 421.11(28.50), 325.04(100.00), 245.08(27.22)	hydrolysis				√				4.92E+06 (U)
									hydrogenation								
									glucuronidation								
									sulfation								
M19	8.84	N	C_26_H_29_O_16_	597.1450	597.1448	12.5	−0.286	597.14(93.54), 421.11(77.15), 245.08(20.01), 175.02(20.91), 135.04(9.39), 243.07(7.15), 157.01(2.96)	hydrolysis				√				1.26E+08 (U)
									hydrogenation								
									bis-glucuronidation								
M20	8.94	N	C_26_H_29_O_16_	597.1450	597.1450	12.5	0.015	597.14(100.00), 421.11(58.07), 245.08(14.71), 175.02(13.24), 135.04(6.56), 157.01(2.30), 243.07(2.11)	hydrolysis				√				9.58E+07 (U)
									hydrogenation								
									bis-glucuronidation								
M21	8.96	N	C_14_H_15_O_8_	311.0761	311.0767	7.5	1.659	113.02(100.00), 175.02(22.38), 135.04(8.38), 311.08(8.25)	isomer of 6-(4-Ethenyl-2-hydroxyphenoxy)-3,4,5-trihydroxyoxane-2-carboxylic acid				√				1.10E+07 (U)
M22	9.19	N	C_20_H_21_O_10_	421.1129	421.1131	10.5	0.491	421.11(100.00), 175.02(19.29), 245.08(8.78), 135.04(8.06)	hydrolysis	√			√				2.52E+09 (U)
									hydrogenation								
									glucuronidation								
M23	9.25	N	C_26_H_27_O_16_	595.1294	595.1292	13.5	−0.355	419.10(100.00), 243.06(44.01), 595.13(36.57), 225.05(8.28), 175.02(7.61)	hydrolysis			√					3.85E+06 (PA)
									bis-glucuronidation								
M24	9.28	N	C_20_H_19_O_13_S	499.0541	499.0541	11.5	−0.075	243.07(100.00), 323.02(87.60), 499.05(81.02), 419.10(17.67), 225.05(10.32)	hydrolysis	√	√	√					2.94E+05 (PM)
									glucuronidation								
									sulfation								
M25	9.29	N	C_26_H_29_O_15_	581.1501	581.1500	12.5	−0.132	581.15(100.00), 243.07(75.56), 405.12(3.07), 225.05(7.90)	hydrolysis	√	√	√					7.62E+06 (PS)
									glucuronidation								
M26	9.31	N	C_26_H_27_O_16_	595.1294	595.1295	13.5	0.166	419.10(100.00), 243.07(53.02), 595.13(36.30), 175.02(9.17), 225.05(6.28)	hydrolysis	√	√	√	√				3.97E+08 (U)
									bis-glucuronidation								
M27	9.35	N	C_26_H_31_O_14_	567.1708	567.1707	11.5	−0.321	243.07(100.00), 405.12(47.37), 225.05(10.26), 199.07(5.95), 175.07(4.86), 567.17(3.04)	isomer of Mulberroside A	√	√						2.20E+06 (PS)
M28	9.35	N	C_26_H_33_O_18_S	665.1382	665.1390	10.5	1.156	96.96(100.00), 243.07(8.96), 405.12(5.44), 665.15(4.77), 567.17(1.44)	hydroxylation						√		3.60E+06 (L)
									hydrogenation								
									Sulfation								
M29	9.35	N	C_27_H_33_O_16_	613.1763	613.1757	11.5	−0.964	243.07(100.00), 405.12(44.79), 567.17(14.36), 225.05(6.33), 199.08(4.35)	dihydroxylation	√	√	√					1.64E+06 (PS)
									methylation								
M30	9.37	N	C_20_H_21_O_13_S	501.0697	501.0700	10.5	0.603	501.07(61.80), 421.11(22.27), 325.04(100.00), 245.08(24.44)	hydrolysis				√				7.60E+07 (U)
									hydrogenation								
									glucuronidation								
									Sulfation								
M31	9.46	N	C_20_H_21_O_12_S	485.0748	485.0748	10.5	-0.048	485.07(87.69), 405.12(2.81), 323.02(7.96), 243.07(100.00), 225.05(11.22), 175.07(6.22), 199.07(5.45), 181.06(4.47)	hydrolysis	√			√				1.29E+06 (U)
									Sulfation								
M32	9.54	N	C_20_H_21_O_13_S	501.0697	501.0700	10.5	0.424	501.07(58.15), 421.11(23.10), 325.04(100.00), 245.08(25.85)	hydrolysis				√				6.59E+07 (U)
									hydrogenation								
									glucuronidation								
									sulfation								
M33	9.61	N	C_20_H_19_O_13_S	499.0541	499.0545	11.5	0.826	243.06(100.00), 323.02(84.23), 499.05(71.06), 419.10(30.24), 225.05(5.80)	hydrolysis		√	√					2.70E+05 (PM)
									glucuronidation								
									sulfation								
M34	9.68	N	C_20_H_19_O_10_	419.0973	419.0973	11.5	-0.031	419.10(100.00), 243.06(51.26), 175.02(17.18), 225.05(11.88), 199.07(9.79)	hydrolysis	√					√		5.51E+07 (PS)
									glucuronidation								
M35	9.72	N	C_20_H_21_O_13_S	501.0697	501.0698	10.5	0.044	501.07(62.29), 421.11(33.75), 325.04(100.00), 245.08(26.83)	hydrolysis				√				2.74E+07 (U)
									hydrogenation								
									glucuronidation								
									sulfation								
M36	9.80	N	C_20_H_19_O_13_S	499.0541	499.0544	11.5	0.526	243.07(100.00), 323.02(86.92), 499.05(39.50), 419.10(18.18), 225.05(2.46)	hydrolysis		√	√					1.01E+06 (PM)
									glucuronidation								
									sulfation								
M37	9.82	N	C_20_H_21_O_10_	421.1129	421.1129	10.5	-0.008	421.11(100.00), 175.02(20.18), 245.08(8.93), 243.07(2.49)	hydrolysis	√			√				7.85E+09 (U)
									hydrogenation								
									glucuronidation								
M38	9.83	N	C_20_H_19_O_9_	403.1024	403.1034	11.5	2.633	403.20(100.00), 245.08(8.58), 135.04(7.70), 175.02(3.61)	hydrolysis				√				5.32E+06 (U)
									dehydroxylation								
									glucuronidation								
M39	9.83	N	C_20_H_19_O_11_	435.0922	435.0913	11.5	-2.017	193.03(100.00), 175.02(5.76), 259.06(4.62), 157.01(3.55), 435.09(2.37)	Hydrolysis				√	√	√		2.14E+07 (U)
									hydroxylation								
									glucuronidation								
M40	9.83	N	C_20_H_21_O_11_	437.1078	437.1073	10.5	-1.230	437.11(100.00), 261.08(36.71), 175.02(20.83), 243.07(6.55)	Hydrolysis				√				1.93E+07 (U)
									Hydration								
									glucuronidation								
M41	10.00	N	C_21_H_23_O_10_	435.1286	435.1271	10.5	-3.363	193.03(100.00), 435.14(15.35), 175.02(3.27), 157.01(3.16), 403.18(1.07), 241.10(0.55), 417.18(0.40)	Hydrolysis					√			1.20E+05 (F)
									hydroxylation								
									Methylation								
M42*	10.03	N	C_14_H_13_O_7_S	325.0377	325.0376	8.5	-0.153	325.04(100.00), 245.08(17.31), 135.04(11.00), 243.06(6.19)	hydrolysis	√	√		√				3.70E+07 (U)
									hydrogenation								
									sulfation								
M43	10.07	N	C_20_H_19_O_10_	419.0973	419.0973	11.5	0.112	419.10(100.00), 243.06(54.06), 175.02(28.47), 199.07(13.35), 225.05(11.16)	hydrolysis	√			√				8.03E+06 (PS)
									glucuronidation								
M44	10.21	N	C_20_H_19_O_10_	419.0973	419.0973	11.5	0.040	419.10(100.00), 243.06(78.98), 175.02(34.79), 225.05(16.89), 199.07(11.06)	hydrolysis	√			√				1.34E+07 (PS)
									glucuronidation								
M45*	10.23	N	C_14_H_13_O_7_S	325.0377	325.0378	8.5	0.400	325.04(100.00), 245.08(19.99), 135.04(12.68), 243.06(5.92)	hydrolysis	√			√				8.45E+07 (U)
									hydrogenation								
									sulfation								
M46	10.28	N	C_20_H_19_O_13_S	499.0541	499.0540	11.5	-0.256	243.07(100.00), 323.02(71.89), 419.10(60.92), 499.05(48.22), 225.05(9.32)	hydrolysis				√				6.55E+06 (U)
									glucuronidation								
									sulfation								
M47	10.44	N	C_20_H_19_O_9_	403.1024	403.1023	11.5	-0.245	403.20(100.00), 227.07(29.95), 175.02(8.58), 113.02(45.99)	hydrolysis	√	√		√		√		2.98E+06 (U)
									dehydroxylation								
									glucuronidation								
M48*	10.48	N	C_14_H_11_O_7_S	323.0220	323.0221	9.5	0.279	243.06(100.00), 323.02(89.74), 225.05(17.97), 199.07(17.37), 175.07(16.90)	hydrolysis	√			√	√			1.44E+07 (PS)
									sulfation								
M49*	10.57	N	C_14_H_9_O_4_	241.0495	241.0490	10.5	-2.096	241.14(100.00), 223.13(31.09), 179.11(19.87), 197.12(11.82)	Hydrolysis	√					√		6.06E+03 (PS)
									Dehydrogenation								
M50*	10.64	N	C_14_H_13_O_4_	245.0808	245.0813	8.5	1.773	203.08(90.41), 123.04(81.08), 245.09(68.29), 227.13(6.47), 159.09(5.34)	Hydrolysis	√							1.05E+06 (PS)
									Hydrogenation								
M51	10.79	N	C_21_H_21_O_10_	433.1129	433.1129	11.5	-0.169	243.06(100.00), 225.05(17.49), 257.08(16.68), 373.09(15.74), 433.11(13.36), 403.10(1.65)	Hydrolysis	√							2.82E+06 (PS)
									Methylation								
									Glucuronidation								
M52	10.84	N	C_20_H_21_O_9_	405.1180	405.1180	10.5	-0.145	405.12(46.47), 229.09(32.19), 175.02(32.02),157.01(4.20), 225.15(1.84)	Oxyresveratrol-4-O-β-D-glucopyranoside	√	√	√	√	√		√	1.98E+08 (U)
M53	10.90	N	C_27_H_31_O_14_	579.1708	579.1706	12.5	-0.418	271.06(100.00), 579.17(35.34), 227.07(2.63), 175.00(2.47)	dehydrogenation	√							7.60E+05 (PS)
									methylation								
M54	10.92	N	C_28_H_33_O_16_	625.1763	625.1758	12.5	-0.850	271.06(100.00), 579.17(49.37), 300.03(7.16), 315.05(3.06), 330.07(2.99)	hydroxylation						√		3.41E+07 (L)
									acetylation								
M55	10.96	N	C_21_H_21_O_10_	433.1129	433.1127	11.5	-0.585	243.06(94.52), 433.11(80.16), 257.08(60.10), 175.02(27.25), 373.09(8.98)	hydrolysis	√					√		7.89E+05 (PS)
									methylation								
									glucuronidation								
M56	11.14	N	C_14_H_9_O_7_S	321.0064	321.0066	10.5	0.655	241.05(24.05), 321.01(14.51), 124.01(13.46), 243.06(5.76)	hydrolysis	√			√				1.52E+06 (U)
									dehydrogenation								
									sulfation								
M57	11.25	N	C_28_H_33_O_15_	609.1814	609.1805	12.5	-1.505	301.07(100.00), 609.18(18.73), 286.05(10.54), 242.06(6.48), 463.12(0.26)	acetylation							√	2.38E+08 (LM)
M58	11.35	N	C_20_H_19_O_9_	403.1024	403.1018	11.5	-1.286	113.02(100.00), 403.10(37.33), 227.07(52.42), 175.02(47.17)	hydrolysis	√		√		√			2.25E+06 (PS)
									dehydroxylation								
									glucuronidation								
M59	11.38	N	C_20_H_21_O_9_	405.1180	405.1177	10.5	-0.811	405.12(100.00), 113.02(53.69), 229.09(45.46), 175.02(16.66)	Oxyresveratrol-3′-O-β-D-glucopyranoside	√			√	√		√	8.43E+07 (U)
M60	11.39	N	C_28_H_35_O_14_	595.2021	595.2017	11.5	-0.692	297.10(100.00), 175.02(8.91), 157.01(1.65), 419.09(0.51), 449.32(0.38)	dimethylation	√			√				1.59E+08 (U)
M61*	11.43	N	C_16_H_15_O_4_	271.0965	271.0964	9.5	-0.278	271.15(100.00), 225.11(14.23), 197.12(7.25), 167.11(32.05), 137.10(10.95)	hydrolysis					√			7.56E+05 (F)
									dimethylation								
M62	11.54	N	C_20_H_23_O_10_	423.1286	423.1283	9.5	-0.717	423.27(100.00), 175.02(29.80), 201.02(28.97), 247.10(16.94), 263.17(3.76)	hydrolysis	√							2.00E+06 (PS)
									hydrogenation								
									hydroxylation								
M63	11.85	N	C_20_H_23_O_9_	407.1337	407.1353	9.5	4.056	407.17(91.79), 201.11(7.99), 137.10(2.85), 227.13(1.82), 245.19(0.70)	hydrolysis					√	√		1.34E+06 (L)
									hydrogenation								
M64	12.06	N	C_20_H_23_O_10_	423.1286	423.1285	9.5	-0.291	423.27(100.00), 421.26(45.89), 175.02(9.52), 405.26(6.22)	Hydrolysis	√				√			1.82E+06 (PS)
									hydrogenation								
									hydroxylation								
M65	12.28	N	C_21_H_21_O_10_	433.1129	433.1126	11.5	-0.862	257.08(100.00), 433.11(86.14), 175.02(28.10), 242.06(1.94)	Hydrolysis	√	√	√	√	√	√		2.62E+07 (PS)
									methylation								
									glucuronidation								
M66	13.01	N	C_20_H_23_O_10_	423.1286	423.1286	9.5	-0.008	423.27(100.00), 421.26(18.72), 175.02(2.65), 405.26(0.98)	Hydrolysis	√			√				3.23E+06 (PS)
									hydrogenation								
									hydroxylation								
M67	13.13	N	C_26_H_33_O_14_	569.1865	569.1871	10.5	1.138	569.33(100.00), 407.28(17.04), 389.27(4.56), 551.32(1.21)	hydrogenation					√			7.90E+04 (F)
M68	13.19	N	C_20_H_23_O_10_	423.1286	423.1282	9.5	-0.953	423.14(100.00), 421.26(85.61), 247.13(14.76), 175.02(8.46), 245.12(2.36)	hydrolysis hydrogenation hydroxylation				√				3.74E+04 (U)
M69	13.63	N	C_14_H_11_O_8_S	339.0169	339.0176	9.5	1.963	339.18(100.00), 259.11(11.96)	hydrolysis				√				2.64E+04 (U)
									hydroxylation								
									sulfation								
M70*	14.30	N	C_14_H_11_O_4_	243.0652	243.0651	9.5	-0.392	243.17(100.00), 199.18(31.85), 225.05(2.25), 201.05(1.38)	Oxyresveratrol	√	√		√			√	1.80E+06 (PS)
M71	14.70	N	C_20_H_23_O_10_	423.1286	423.1282	9.5	-0.882	423.27(100.00), 407.28(10.68), 421.26(5.80), 405.26(4.50)	hydrolysis				√				5.85E+04 (U)
									hydrogenation								
									hydroxylation								

PS, plasma treated by SPE; PM, plasma treated by methanol; PA, plasma treated by acetonitrile; U, urine; F, feces; L, liver tissue; LM, liver microsome; √, detected; *, components in Table 2.

#### 3.3.1 Identification of mulberroside A metabolites

**M0** was identified as the prototype of mulberroside A with the [M-H]^-^ ions at *m/z* 567.1707 (C_26_H_31_O_14_, -0.197 ppm) in negative ion modes. The fragment ions at *m/z* 243, *m/z* 567, *m/z* 405, *m/z* 225 were detected, which were consistent with the mass fragmentation behavior of the reference substance of mulberroside A. Therefore, **M0** was characterized as the prototype of mulberroside A. **M27** possessed the same theoretical [M-H]^-^ and product ions with **M0**, but compared with **M0**, it was eluted later than **M0**. So, **M27** was deduced as isomer of mulberroside A.

**M1** and **M21** generated [M-H]^-^ ions at *m/z* 285.0607 (C_12_H_13_O_8_, 0.618 ppm) and *m/z* 311.0767 (C_14_H_15_O_8_, 1.659 ppm) respectively. The neutral loss of 176 Da (*m/z* 285→*m/z* 109 and *m/z* 311→*m/z* 135) was generated in their ESI-MS/MS spectra, indicating that their structures contain glucuronic acid (Glu A). In addition, the product ion at *m/z* 175 ([Glu A-H]^-^) was detected, which also proved the existence of Glu A. According to pubchem database (https://pubchem.ncbi.nlm.nih.gov/) and molecular formulas, **M1** was identified as β-D-Glucopyranosiduronic Acid-3-Hydroxyphenyl and **M21** was characterized as isomer of 6-(4-Ethenyl-2-hydroxyphenoxy)-3,4,5-trihydroxyoxane-2-carboxylic acid. They were the glucuronidation metabolites of compounds formed by the rupture of C1-α bond or C1′-β bond in mulberroside A.

**M2** and **M6** possessed the same theoretical [M-H]^-^ ions at *m/z* 583.1652 (C_26_H_31_O_15_, mass error within 2 ppm) with the retention time of 7.72 and 8.20 min, respectively. They were 16 Da more massive than **M0**, which suggested that they might be the hydroxylation products of mulberroside A. The fragment ion at *m/z* 259 ([M-H-2Glu]^-^) was 16 Da more massive than *m/z* 243, providing evidence for hydroxylation reaction. The product ions at *m/z* 421 ([M-H-Glu]^-^) was formed by losing Glu. Therefore, it was speculated that **M2** and **M6** were hydroxylation products of mulberroside A.

**M7** was eluted at 8.20 min with the [M-H]^-^ at *m/z* 599.1602 (C_26_H_31_O_16_, -0.736 ppm). It was 32 Da (2O) more massive than **M0**, indicating that it might be the dihydroxylation product of mulberroside A. The fragment ions involved *m/z* 137, *m/z* 599, *m/z* 125, *m/z* 437, and *m/z* 275. The [M-H]^-^ ion lost Glu to form *m/z* 437 ([M-H-Glu]^-^), and lost another Glu to form *m/z* 275 ([M-H-2Glu]^-^). We first assumed that two benzene rings were each bound to a hydroxyl group. Then it was split by Chem Draw, and we found that the α-β bond of the product ion at *m/z* 275 ([M-H-2Glu]^-^) broke to form C_7_H_5_O_3_^2-^, which molecular weight was exactly *m/z* 137. From these, it was speculated that **M7** was dihydroxylation product of mulberroside A and two hydroxyl groups were bonded to the benzene rings on both sides.

**M5** and **M29**, with the molecular formula of C_27_H_33_O_16_, showed the same theoretical [M-H]^-^ ions at *m/z* 613.1763 and they were 46 Da (CH_2_O_2_) more massive than **M0**. The loss of 30 Da (*m/z* 435→*m/z* 405) suggested the existence of methoxy. In addition, the product ions at *m/z* 243, *m/z* 405, *m/z* 567 were representative ions of mulberroside A. As a consequence, it was deemed that **M5** and **M29** were dihydroxylation and methylation products of mulberroside A.

**M9** eluted at 8.24 min and generated the [M-H]^-^ at *m/z* 601.1769 (C_26_H_33_O_16_, 1.046 ppm). It was 34 Da (H_2_O_2_) more massive than **M0**, which indicated that it was hydrogenation and hydroxylation product of mulberroside A. The fragment ions at *m/z* 137, *m/z* 243, *m/z* 601, *m/z* 567, *m/z* 439 ([M-H-Glu]^-^) were observed in its ESI-MS/MS spectrum. The α-β bond of **M9** was broken and then lost Glu to form *m/z* 137 (C_7_H_5_O_3_^−^), while *m/z* 243 and *m/z* 567 were characteristic ions of mulberroside A. Therefore, **M9** was identified as vicinal diols formed by dihydroxylation at α-β bond of mulberroside A.

**M54**, with the retention time of 10.92 min, was 58 Da (C_2_H_2_O_2_) more massive than **M0**. The fragment ion at *m/z* 579 was fragmented by losing of CO and H_2_O from [M-H]^-^ ion, while *m/z* 271 was identified as C_12_H_15_O_7_^−^ formed by the fracture of the bonds C-1 and α. The neutral loss of 30 Da (*m/z* 330→*m/z* 300) indicated that methoxy was contained in the structure. Hence, **M54** was finally characterized as hydroxylation and acetylation product of mulberroside A.

**M57** was 42 Da (C_2_H_2_O) more massive than **M0** with the molecular formula of C_28_H_33_O_15_. It was speculated that **M57** was acetylation product of mulberroside A. In the ESI-MS/MS spectrum of **M57**, the fragment ion at *m/z* 463 removed Glu to form *m/z* 301, then lose -CH_3_ to form the ion *m/z* 286. The product ion at *m/z* 297 was 30 Da more massive than *m/z* 267 ([M-H-2Glu]^-^), which proved the existence of methoxy.

**M60** owned the [M-H]^-^ ion at *m/z* 595.2017 (C_28_H_35_O_14_, -0.692 ppm) with the retention time of 11.39 min and was 28 Da (C_2_H_4_) more massive than **M0**. We speculated that **M60** was dimethylation product of mulberroside A. In its ESI-MS/MS spectrum, the loss of 30 Da (*m/z* 449→*m/z* 419) was detected, which confirmed our prediction. In addition, the fragment ion at *m/z* 297 was identified as C_14_H_18_O_7_^−^ formed by the rupture of α-β bond.

**M67** was 2 Da more massive than **M0** and generated the [M-H]^-^ at *m/z* 569.1871 (C_26_H_33_O_14_, 1.138 ppm) with the retention time of 13.13 min, which indicated that it might be hydrogenation product of mulberroside A. The product ion at *m/z* 407 ([M-H-Glu]^-^) was 2 Da more massive than that of *m/z* 405. Thus, **M67** was ultimately characterized as hydrogenation product of mulberroside A.

Metabolites **M4** and **M28**, with the [M-H]^-^ ions at *m/z* 665.1384 (0.239 ppm) and *m/z* 665.1390 (1.156 ppm) respectively, was 98 Da more massive than **M0**, suggesting that they might be the hydrogenation, hydroxylation and sulfation metabolite of mulberroside A. The fragment ions at *m/z* 96.96 ([SO_4_H]^-^) had the highest abundance, which was a base peak, indicating that S may be contained. In addition, *m/z* 243, *m/z* 567, *m/z* 405 were characteristic fragments of mulberroside A. Therefore, it was speculated that **M4** and **M28** were hydrogenation, hydroxylation and sulfation products of mulberroside A.

**M52**, **M59** and **M70** generated the [M-H]^-^ ions at *m/z* 405.1180 (-0.145 ppm), *m/z* 405.1177 (-0.811 ppm), and *m/z* 243.0651 (-0.392 ppm), respectively. **M52** and **M59** were 162 Da less massive than **M0** while **M70** was 324 Da less massive than **M0**. Moreover, the product ion at *m/z* 225 was included in their ESI-MS/MS spectra and *m/z* 199 was detected in the ESI-MS/MS spectra of **M70**. It was speculated that they were hydrolysates of mulberroside A. **M52** and **M59** were formed by losing a molecule of Glu while **M70** was formed by losing two molecules of Glu. In the experiment of Zhaxi et al., they isolated three metabolites of mulberroside A from rat intestinal contents and feces. The three metabolites were identified as oxyresveratrol-2-O-β-D-glucopyranoside, oxyresveratrol-3′-O-β-D-glucopyranoside and oxyresveratrol according to ESI-MS spectrum, ^1^H and ^13^C-NMR spectroscopy (Zhaxi et al., 2010). In their study, oxyresveratrol-3′-O-β-D-glucopyranoside (31.3 min) was eluted later than oxyresveratrol-2-O-β-D-glucopyranoside (27.8 min). However, the hydrolysis products of mulberroside A that formed by losing a Glu should be oxyresveratrol-4-O-β-D-glucopyranoside and oxyresveratrol-3′-O-β-D-glucopyranoside. Hence, it was speculated that oxyresveratrol-2-O-β-D-glucopyranoside was formed by oxyresveratrol combined with a Glu. Therefore, in our experiment, **M52**, **M59** were deduced as oxyresveratrol-4-O-β-D-glucopyranoside and oxyresveratrol-3′-O-β-D-glucopyranoside based on the retention time. Furthermore, **M70** was identified as oxyresveratrol according to the literature (Xu et al., 2014).

**M53** was deemed as the dehydrogenation and methylation product of mulberroside A, with the [M-H]^-^ ion at *m/z* 579.1706 (C_27_H_31_O_14_, -0.418 ppm), which was 12 Da more massive than **M0**. The DPI at *m/z* 271 was deduced as C_12_H_15_O_7_^−^ formed by the rupture of α-β bond.

#### 3.3.2 Identification of oxyresveratrol-4-O-β-D-glucopyranoside or oxyresveratrol-3′-O-β-D-glucopyranoside metabolites

**M3, M16** and **M25**, with the retention time of 7.98, 8.77 and 9.29 min, possessed the same theoretical [M-H]^-^ ion at *m/z* 581.1501 (C_26_H_29_O_15_, mass error within 1.00 ppm). They were 176 Da more massive than **M52** or **M59**, which suggested that they may be the glucuronidation products of **M52** or **M59**. In the ESI-MS/MS spectrum, the product ions at *m/z* 243 ([M-H-Glu A-Glu]^-^), *m/z* 405 ([M-H-Glu A]^-^), *m/z* 225 ([M-H-Glu A-Glu-H_2_O]^-^) were detected. Therefore, **M3**, **M16** and **M25** were identified as glucuronidation metabolites of oxyresveratrol-4-O-β-D-glucopyranoside or oxyresveratrol-3′-O-β-D-glucopyranoside.

**M8**, detected at 8.20 min, generated the [M-H]^-^ ion at *m/z* 437.1079 (C_20_H_21_O_11_, 0.042 ppm) and was 32 Da more massive than **M52** or **M59**, indicating that it may be the dihydroxylation metabolite of oxyresveratrol-4-O-β-D-glucopyranoside or oxyresveratrol-3′-O-β-D-glucopyranoside. The fragment ions at *m/z* 275 ([M-H-Glu]^-^) and *m/z* 257 ([M-H-Glu-H_2_O]^-^) were detected in its ESI-MS/MS spectrum. In addition, the fragment of *m/z* 137 was also detected, which was presumed to be C_7_H_6_O_3_^−^ formed by α-β bond breakage.

**M13** eluted at 8.57 min, was deduced as hydrogenation and glucuronidation product of **M52** or **M59**. The successive loss of 162 and 176 Da (*m/z* 583→*m/z* 421→*m/z* 245) indicated that Glu and Glu A may be contained. Besides, the DPI at *m/z* 245 ([M-H-Glu-Glu A]^-^) was 2 Da more massive than *m/z* 243 (characteristic ion of **M52** and **M59**). Eventually, **M13** was identified as hydrogenation and glucuronidation metabolite of oxyresveratrol-4-O-β-D-glucopyranoside or oxyresveratrol-3′-O-β-D-glucopyranoside. In the ESI-MS/MS spectrum of **M14**, the successive loss of Glu A, Glu and Glu A (*m/z* 759→m/z 583→m/z 421→m/z 245) was detected, which indicated that two molecules of Glu A and a molecule of Glu may be contained. So, **M14** was characterized as hydrogenation and bis-glucuronidation product of oxyresveratrol-4-O-β-D-glucopyranoside or oxyresveratrol-3′-O-β-D-glucopyranoside.

**M31**, with the [M-H]^-^ ion at *m/z* 485.0748 (C_20_H_21_O_12_S, -0.048ppm), was 80 Da more massive than **M52** or **M59**, which implied that it might be the sulfation product. In its ESI-MS/MS spectrum, the product ions at *m/z* 405 ([M-H-SO_3_]^-^) and *m/z* 243 ([M-H-SO_3_-Glu]^-^) confirmed the metabolite identification. Hence, **M31** was tentatively characterized as sulfation product of oxyresveratrol-4-O-β-D-glucopyranoside or oxyresveratrol-3′-O-β-D-glucopyranoside.

**M63** was 2 Da more massive than **M52** or **M59**, indicating that they were hydrogenation metabolites of **M52** or **M59**. The fragment ion at *m/z* 201 were detected in its ESI-MS/MS spectrums, which was formed by the successive loss of Glu and CO_2_. In addition, the product ion at *m/z* 227 ([M-H-Glu-H_2_O]^-^) was also detected.

**M41** possessed the [M-H]^-^ ion at *m/z* 435.1271 (C_21_H_23_O_10_, -3.363ppm), with the retention time of 10.00 min. The DPIs at *m/z* 417 [M-H-H_2_O]^-^, *m/z* 403 and *m/z* 193 were generated in its ESI-MS/MS spectrum. Therefore, it was speculated that **M41** was hydroxylation and methylation metabolite of oxyresveratrol-4-O-β-D-glucopyranoside or oxyresveratrol-3′-O-β-D-glucopyranoside. The fragment of *m/z* 403 was presumed to be formed by the loss of H_2_O from *m/z* 421 (*m/z* 405+16) and *m/z* 193 may be C_11_H_14_O_3_^−^ formed by benzene ring opening.

**M62**, **M64**, **M66**, **M68** and **M71**, with the same molecular formula of C_20_H_23_O_10_, had the same theoretical [M-H]^-^ ion at *m/z* 423.1286. They were 18 Da more massive than **M52** or **M59**, suggesting that they may be hydrogenation and hydroxylation products of **M52** or **M59**. The DPIs at *m/z* 405, *m/z* 201, *m/z* 263 and *m/z* 247 were detected, which provided evidence for the metabolite identification.

#### 3.3.3 Identification of oxyresveratrol metabolites

**M17**, **M24**, **M33**, **M36** and **M46** were 256 Da more massive than **M70** with the same theoretical [M-H]^-^ ions at *m/z* 499.0541 (C_20_H_19_O_13_S, error<1.00 ppm). In the ESI-MS/MS spectra, the successive loss of Glu A and SO_3_ (*m/z* 499 [M-H]^-^→*m/z* 419 [M-H-SO_3_]^-^→*m/z* 243 [M-H-SO_3_-Glu A]^-^, *m/z* 499 [M-H]^-^→*m/z* 323 [M-H-Glu A]^-^→*m/z* 243 [M-H-Glu A-SO_3_]^-^) was observed. Therefore, they were eventually characterized as glucuronidation and sulfation products of oxyresveratrol.

**M34**, **M43** and **M44** were tentatively identified as glucuronidation products of **M70** according to the neutral loss of 176 Da (*m/z* 419→*m/z* 243). In addition, the fragment ion at *m/z* 175 ([Glu A-H]^-^) provided evidence for the existence of glucuronic acid group. **M48** was 80 Da more massive than **M70**, with the [M-H]^-^ ion at *m/z* 323.02209 (C_14_H_11_O_7_S, 0.279 ppm), implying that it may be sulfation product of **M70**. The neutral loss of 80 Da (*m/z* 323→*m/z* 243) proved the existence of SO_3_ in its ESI-MS/MS spectrum.

**M50** was 2 Da more massive than **M70** and it was preliminarily deduced as the hydrogenation metabolite of oxyresveratrol. It was produced by hydrogenation reduction reaction occur at the α-β bond according to the product ion at *m/z* 123, which was formed by the rupture of α-β bond. The fragment ions at *m/z* 203 ([[M-H-CO-CH_2_]^−^]^-^) and *m/z* 159 ([M-H-C_3_O_2_-H_2_O]^−^) were generated in its ESI-MS/MS spectra. Besides, the DPI at *m/z* 227 ([M-H-H_2_O]^-^) was also detected. **M49** was 2 Da less massive than **M70**, indicating that it might be the dehydrogenation product of oxyresveratrol. The DPIs at *m/z* 197 ([M-H-CO_2_]^-^), *m/z* 179 ([M-H-CO_2_-H_2_O]^-^) and *m/z* 223 ([M-H-H_2_O]^-^) provided evidence for the metabolite identification. Finally, **M50** was identified as hydrogenation metabolite of oxyresveratrol and **M49** was characterized as dehydrogenation product of oxyresveratrol.

**M10**, **M51**, **M55** and **M65**, with the same theoretical [M-H]^-^ ion at *m/z* 433.1129 (C_21_H_21_O_10_, error ≤1.00 ppm), were 190 Da more massive than **M70**. The DPI at *m/z* 257 ([M-H-Glu A]^-^) and the continuous neutral loss of 30 Da (*m/z* 433→*m/z* 403→*m/z* 373) were detected, suggesting that Glu A and -OCH_3_ might be contained. Thus, they were eventually deduced as methylation and glucuronidation metabolites of oxyresveratrol.

**M11**, **M19** and **M20**, eluted at 8.43 min, 8.84 min and 8.94 min respectively, were deduced as hydrogenation and bis-glucuronidation products of **M70**. In the ESI-MS/MS spectra, the successive loss of 176 Da (*m/z* 597→*m/z* 421→*m/z* 245) indicated that two molecules of Glu A might be contained. Furthermore, the DPI at *m/z* 175 confirmed the existence of Glu A and the DPI at *m/z* 245 ([M-H-2Glu A]^-^) was 2 Da more massive than *m/z* 243 ([M-H]^-^ ion of **M70**). Therefore, they were eventually identified as hydrogenation and glucuronidation products of oxyresveratrol.

**M12**, eluted at 8.52 min, was 174 Da more massive than **M70**. The neutral loss of 176 Da (*m/z* 421→*m/z* 245 and *m/z* 417→*m/z* 241) was detected in both ESI-MS/MS spectra, which was suggested that Glu A might be contained in its structure. What’s more, the DPI at *m/z* 241 of **M12** was 2 Da less massive than *m/z* 243 ([M-H]^-^ ion of M70). Thus, **M12** was characterized as dehydrogenation and glucuronidation metabolite of oxyresveratrol. **M22** and **M37**, with the retention time of 9.19 and 9.82 min, were 178 Da more massive than M70. The neutral loss of 176 Da (*m/z* 421→*m/z* 245 and *m/z* 417→*m/z* 241) provided evidence for the existence of Glu A. In addition, the DPI at *m/z* 245 was 2 Da more massive than *m/z* 243. Finally, they were deduced as hydrogenation and glucuronidation product of oxyresveratrol.

**M15**, **M23** and **M26** were identified as bis-glucuronidation products of **M70** according to the successive loss of Glu A (*m/z* 595→*m/z* 419→*m/z* 243) with the retention time of 8.76 min, 9.25 min and 9.31 min, respectively. **M18**, **M30**, **M32** and **M35**, with the molecular formula of C_20_H_21_O_13_S, generated the same theoretical [M-H]^-^ ions at *m/z* 501.0697. The successive loss of 176 Da (*m/z* 421→*m/z* 245) and 80 Da (*m/z* 501→*m/z* 421, *m/z* 325→*m/z* 245) was detected in their ESI-MS/MS spectra, indicating that they might be the glucuronidation and sulfation products of **M70**. In addition, the DPI at *m/z* 245 was 2 Da more massive than the [M-H]^-^ ion of **M70** (*m/z* 243). So, they were deduced as the hydrogenation, glucuronidation and sulfation products of oxyresveratrol.

**M38**, **M47** and **M58** were deduced as the dehydroxylation and glucuronidation metabolites of oxyresveratrol, with the molecular formula of C_20_H_19_O_9_. The DPIs at *m/z* 227 [M-H-Glu A]^-^ (*m/z* 403–176 Da, *m/z* 243–16 Da) provided the evidence for the metabolite identification. The successive loss of 176 and 16 Da (*m/z* 435→*m/z* 259→*m/z* 243) was observed in the ESI-MS/MS spectrum of **M39**, indicating that Glu A and -OH may be contained. Therefore, **M39** was identified as hydroxylation and glucuronidation product of oxyresveratrol. **M40** was characterized as hydration and glucuronidation metabolite of oxyresveratrol based on the successive loss of Glu A and H_2_O (*m/z* 437→*m/z* 261→*m/z* 243).

**M42** and **M45** were deemed to be the hydrogenation and sulfation products of oxyresveratrol while **M56** was identified as dehydrogenation and sulfation metabolite of oxyresveratrol. The DPIs at *m/z* 245 (*m/z* 325–80 Da, *m/z* 243+2 Da) and *m/z* 241 (*m/z* 321–80 Da, *m/z* 243–2 Da) provided the evidence for the metabolite identification.

**M61** possessed the [M-H]^-^ ion at *m/z* 271.0964 (C_16_H_15_O_4_, -0.278 ppm), which was 28 Da (C_2_H_4_) more massive than **M70**. It was presumed to be the dimethylation product of oxyresveratrol. The successive loss of 30 Da (*m/z* 197→*m/z* 167→*m/z* 137) provided evidence for the existence of methoxy.

**M69**, with the [M-H]^-^ ion at *m/z* 339.0176 (C_14_H_11_O_8_S, 1.963 ppm), was eluted at 13.63 min. The neutral loss of 80 Da (*m/z* 339→*m/z* 259) was observed in the ESI-MS/MS spectrum, indicating that SO_3_ might be contained in its structure. Besides, the fragment ion at *m/z* 259 was 16 Da more massive than *m/z* 243, which suggested that it may be hydroxylation product of **M70**. Hence, it was speculated that **M69** was hydroxylation and sulfation product of oxyresveratrol.

### 3.4 Analysis of metabolic reaction of mulberroside A

**FIGURE 6:**
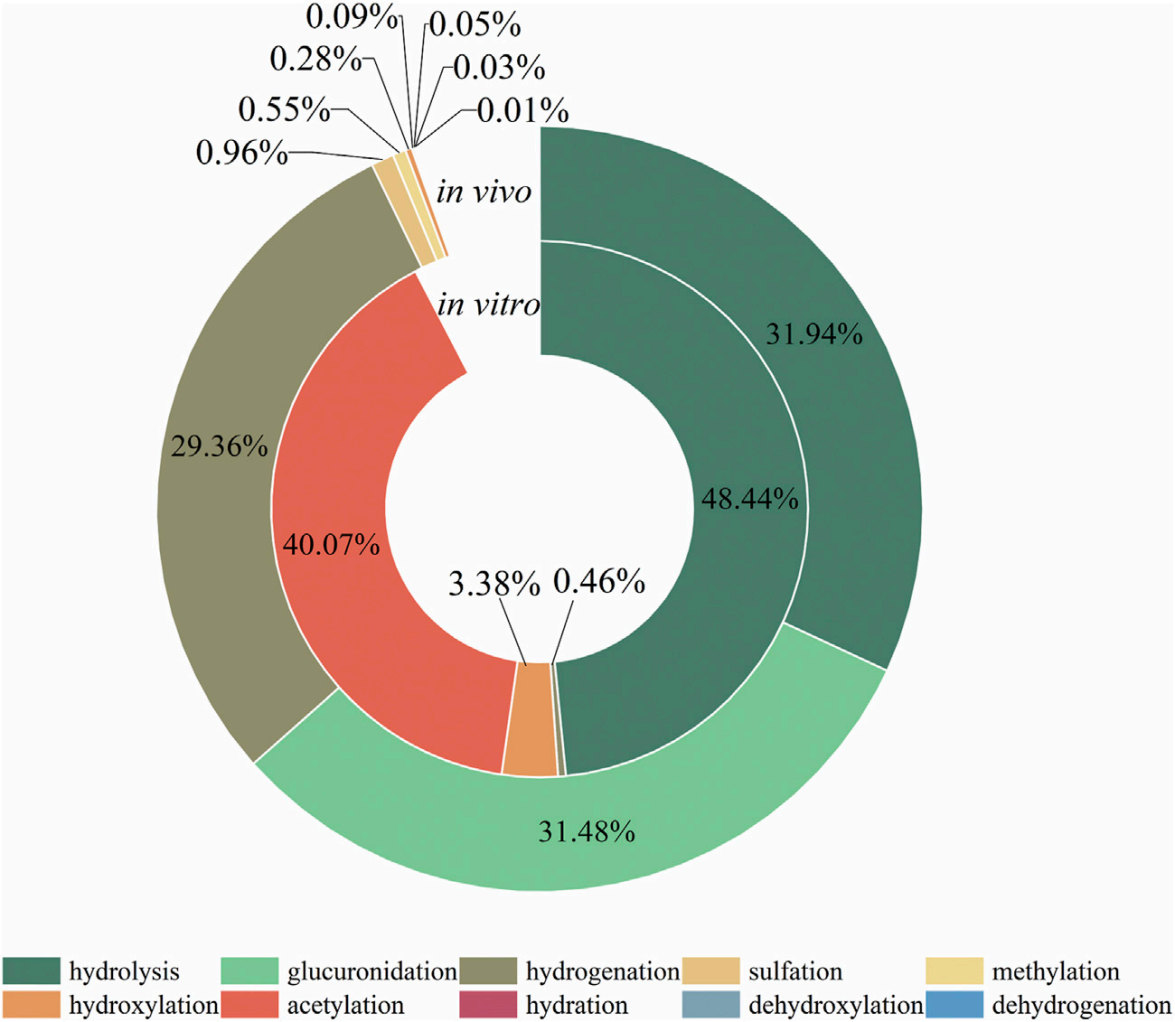
Proportion of the area of each metabolic reaction peak.

The metabolic reactions of mulberroside A *in vivo* and *in vitro* of rats were hydrolysis, hydrogenation, hydroxylation, methylation, acetylation, sulfation, glucuronidation and so on. In this paper, the product peak areas of each type of metabolic reaction were summarized, as shown in Figure 6. The results showed that the metabolic reactions of mulberroside A *in vivo* were mainly hydrolysis, glucuronidation and hydrogenation. The total peak area of hydrolysis metabolites accounted for the largest proportion. Therefore, it was preliminarily speculated that after entering the body, mulberroside A was hydrolyzed into aglycone firstly, and then a series of metabolic reactions occur. While *in vitro*, the most abundant compounds were hydrolysate and acetylation products of mulberroside A.

### 3.5 Analysis of proposed metabolic characteristics of mulberroside A

**FIGURE 7:**
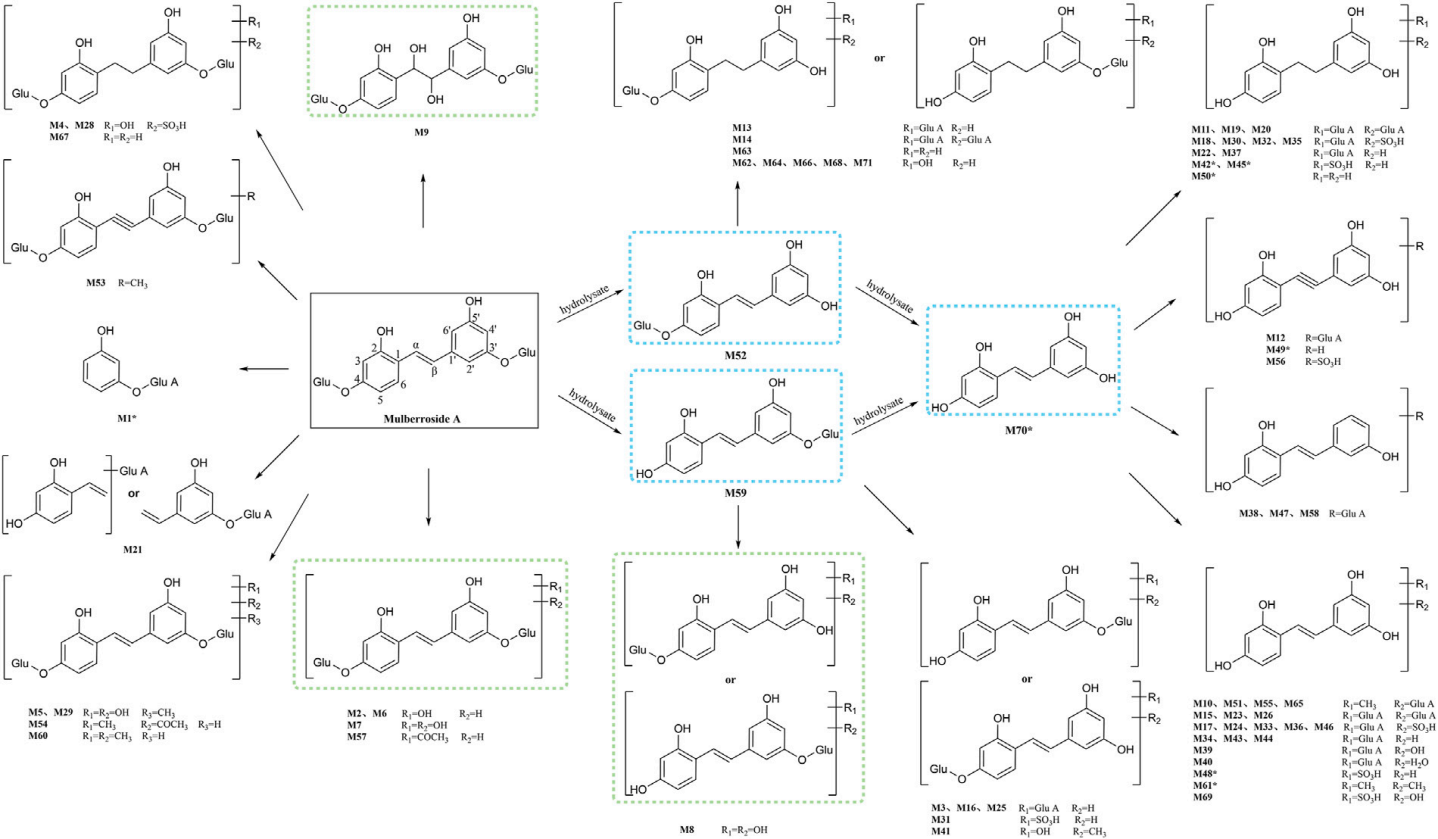
The proposed metabolic pathways of mulberroside A *in vivo* and *in vitro* of rats. Note: Glu: glucose; Glu A: glucuronic acid; component with blue flame: metabolite *in vivo* and *in vitro*; component with green flame: metabolite *in vitro*; *: components in Table 2.

In this experiment, a total of 72 metabolites including mulberroside A were detected, 40 in blood, 37 in urine, 12 in feces, 9 in liver, and 10 in liver microsomes. The prototype component was only detected in rat plasma samples, which indicated that mulberroside A could be absorbed into the blood, and then a series of metabolic reactions occurred. There was no significant difference on the type of metabolites in plasma and urine, and the metabolites in feces were mainly glucuronidation, sulfation and methylation products of oxyresveratrol-4-O-β-D-glucopyranoside or oxyresveratrol-3′-O-β-D-glucopyranoside. It was speculated that mulberroside A was metabolized in 3 pathways *in vivo* and *in vitro* of rats. (1) The metabolic reactions such as sulfation, hydroxylation and methylation were occurred on the basis of mulberroside A (M2, M4, M5, M6, M7, M9, M28, M29, etc.). In this pathway, glucuronidation reaction was undetected. (2) Mulberroside A was hydrolyzed to form oxyresveratrol-4-O-β-D-glucopyranoside (M52) or oxyresveratrol-3′-O-β-D-glucopyranoside (M59), on which metabolic reactions took place, such as M3, M8, M13, M14, M16, M25, M31 and so on. (3) Mulberroside A continued to be hydrolyzed and lose another Glu to produce oxyresveratrol (M70). Then, groups such as glucuronic acid, sulfate and methyl were bound to oxyresveratrol (M10, M11, M15, M17, M18, M19, etc.). Besides, β-D-Glucopyranosiduronic Acid 3-Hydroxyphenyl (M1) and isomer of 6-(4-Ethenyl-2-hydroxyphenoxy)-3,4,5-trihydroxyoxane-2-carboxylic acid (M21) were formed by the rupture of C1′-β bond. The proposed metabolic pathways of mulberroside A are shown in Figure 7.

Furthermore, the *in vivo* and *in vitro* metabolites of mulberroside A were mostly based on its hydrolyzed products oxyresveratrol-4-O-β-D-glucopyranoside or oxyresveratrol-3′-O-β-D-glucopyranoside and oxyresveratrol. The main metabolic reactions were hydrolysis, hydroxylation, methylation, sulfation, glucuronidation and their compound reactions. The metabolic result showed that the main forms of mulberroside A *in vivo* were the glucuronidation and sulfation products of its aglycone, while *in vitro* they were mainly the hydroxylation and hydrolysis products of its prototype. The *in vitro* pharmacokinetic study of mulberroside A also showed that the glucose group on mulberroside A was rapidly hydrolyzed to generate monoglycosides and its aglycone oxyresveratrol when incubated anaerobically with intestinal bacteria (Mei et al., 2012), which was consisted with our findings.

### 3.6 Comparison of three different sample preparation methods

In the study of drug metabolism, deproteinization of biological samples is essential on account of a large number of proteins were contained in plasma, which interferes with the identification of metabolites. At present, the main methods of protein removal are organic solvent precipitation (methanol, acetonitrile, methanol-ethanol, methanol-acetonitrile-acetone, etc.) and solid phase extraction method (Xu et al., 2011). In our experiment, the plasma samples of rats were treated by solid phase extraction, methanol precipitation and acetonitrile precipitation respectively. In the end, 36 metabolites were detected in the plasma samples treated by SPE while 17 metabolites were detected in the plasma samples treated by methanol precipitation and 15 metabolites in the plasma samples treated by acetonitrile precipitation. It was obvious that solid phase extraction method was significantly better than the other two methods, which was consistence with the study results of Liu et al. (Liu et al., 2020). It was speculated that solid phase extraction plays the role of separation, enrichment and concentration in the process of sample pretreatment. On the contrary, biological samples may be diluted by methanol precipitation and acetonitrile precipitation. In addition, the type of organic solvents and the proportion of different solvents also play important roles in the pretreatment of biological samples.

### 3.7 Network pharmacology analysis of mulberroside A and its metabolites

#### 3.7.1 Construction and analysis of metabolite-target network

**FIGURE 8:**
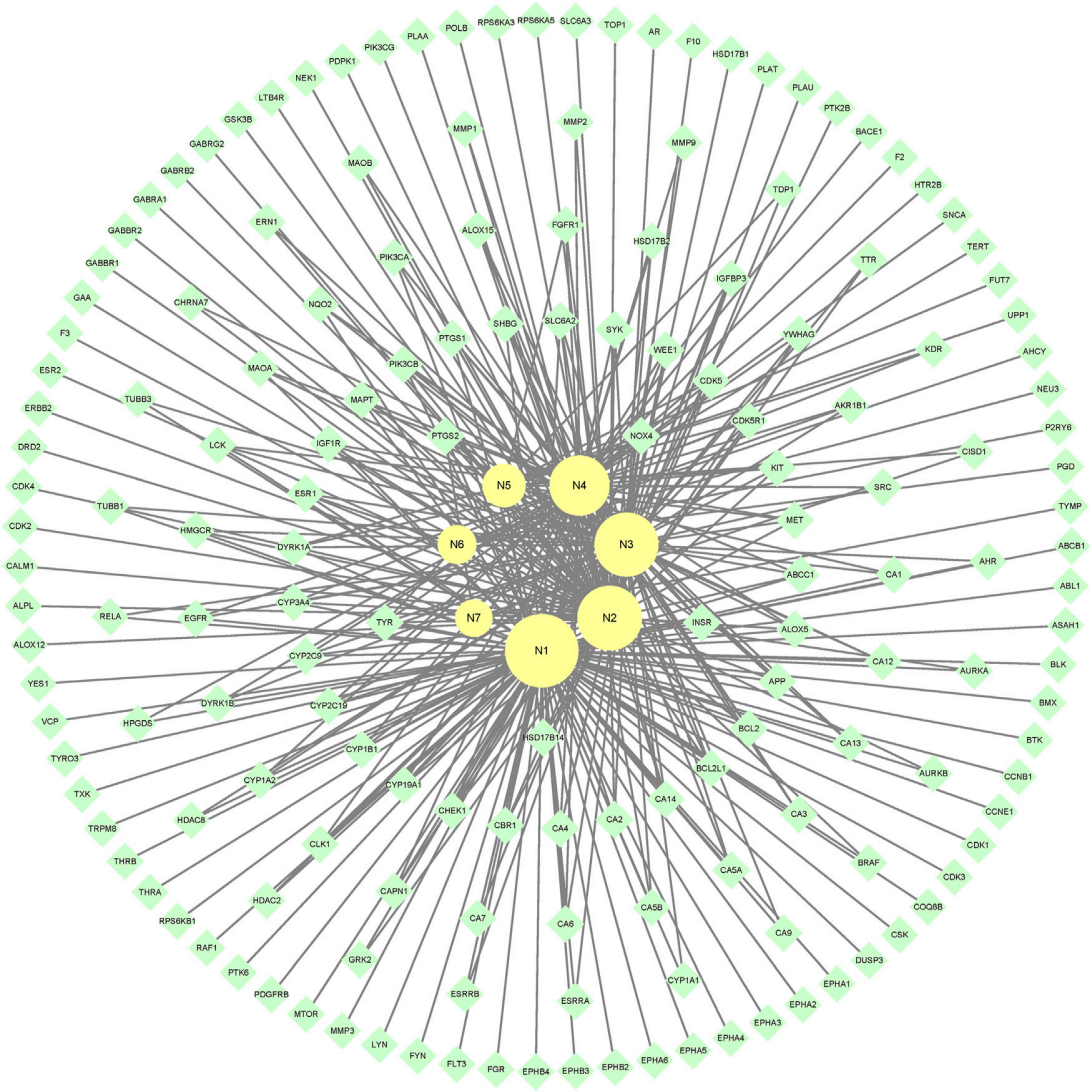
The metabolite-target network of mulberroside A metabolites.

7 metabolites (N1–N7) with high gastrointestinal absorption and drug-likeness were selected by Swiss ADME. The information of the 7 metabolites is shown in Table 2. However, the Swiss ADME results showed that mulberroside A had no drug-likeness and no target of mulberroside A was obtained by Swiss Target Prediction. So, mulberroside A was not included. As a result, a total of 167 targets were obtained using Swiss Target Prediction. Then, the metabolite-target network with 174 nodes and 350 edges was constructed based on the information by Cytoscape 3.8.2, as shown in Figure 8. The area of the component graph in the figure is proportional to the degree value, so it can be seen intuitively that N1 has the largest effect, followed by N2 and N3.

**TABLE 2:** Target information of 7 metabolites of mulberroside A.

No.	Target number	Structure	Identification
N1 (M61)	102	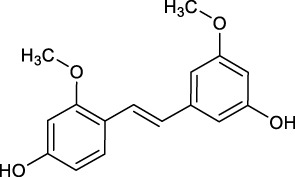	dimethylated product of oxyresveratrol
N2 (M50)	78	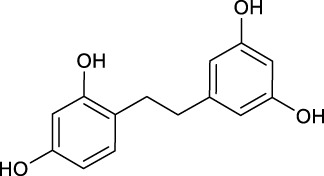	2,4,3′,5′-Tetrahydroxybibenzyl
N3 (M70)	76	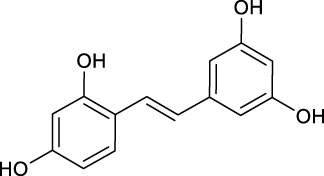	Oxyresveratrol
N4 (M49)	65	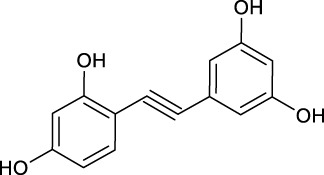	dehydrogenation product of oxyresveratrol
N5 (M42/M45)	19	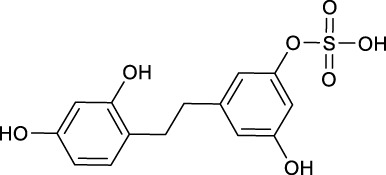	hydrogenation and sulfation product of oxyresveratrol
N6 (M1)	7	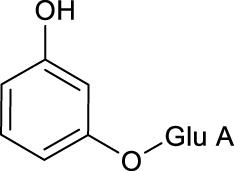	β-D-Glucopyranosiduronic Acid 3-Hydroxyphenyl
N7 (M48)	3	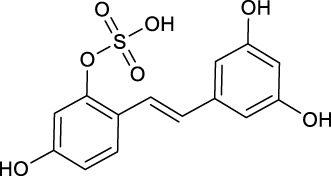	sulfation product of oxyresveratrol

### 3.7.2 GO enrichment analysis of target proteins

**FIGURE 9:**
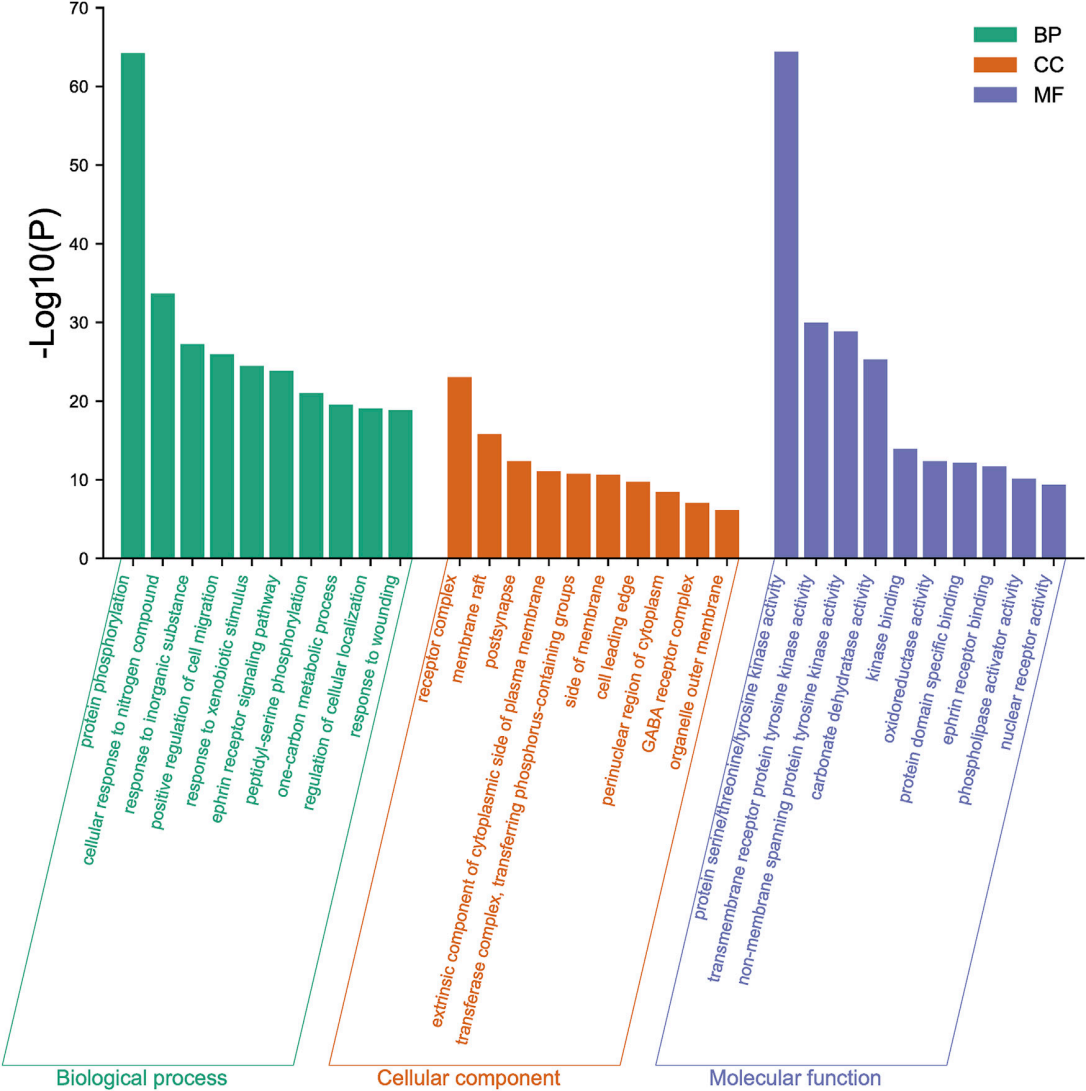
GO enrichment analysis of mulberroside A metabolites.

The 167 targets were submitted to the Metascape for GO enrichment analysis, including molecular functions (MF), biological processes (BP) and cellular components (CC). A total of 1702 GO items were obtained finally (164 of MF, 1427 of BP and 111 of CC) (*p* < 0.01). The top 10 clusters with their representative enriched terms (one per cluster) were selected and they are shown in Figure 9. The serine/threonine/tyrosine kinase activity, transmembrane receptor protein tyrosine kinase activity, non-membrane spanning protein tyrosine kinase, carbonate dehydratase activity and kinase binding were involved in MF. In the aspect of BP, there were mainly protein phosphorylation, cellular response to nitrogen compound, response to inorganic substance, positive regulation of cell migration and response to xenobiotic stimulus. The receptor complex, membrane raft, postsynapse, extrinsic component of cytoplasmic side of plasma membrane and transferase complex, transferring phosphorus-containing groups were mainly included in the category of CC.

### 3.7.3 KEGG analysis of target proteins

**FIGURE 10:**
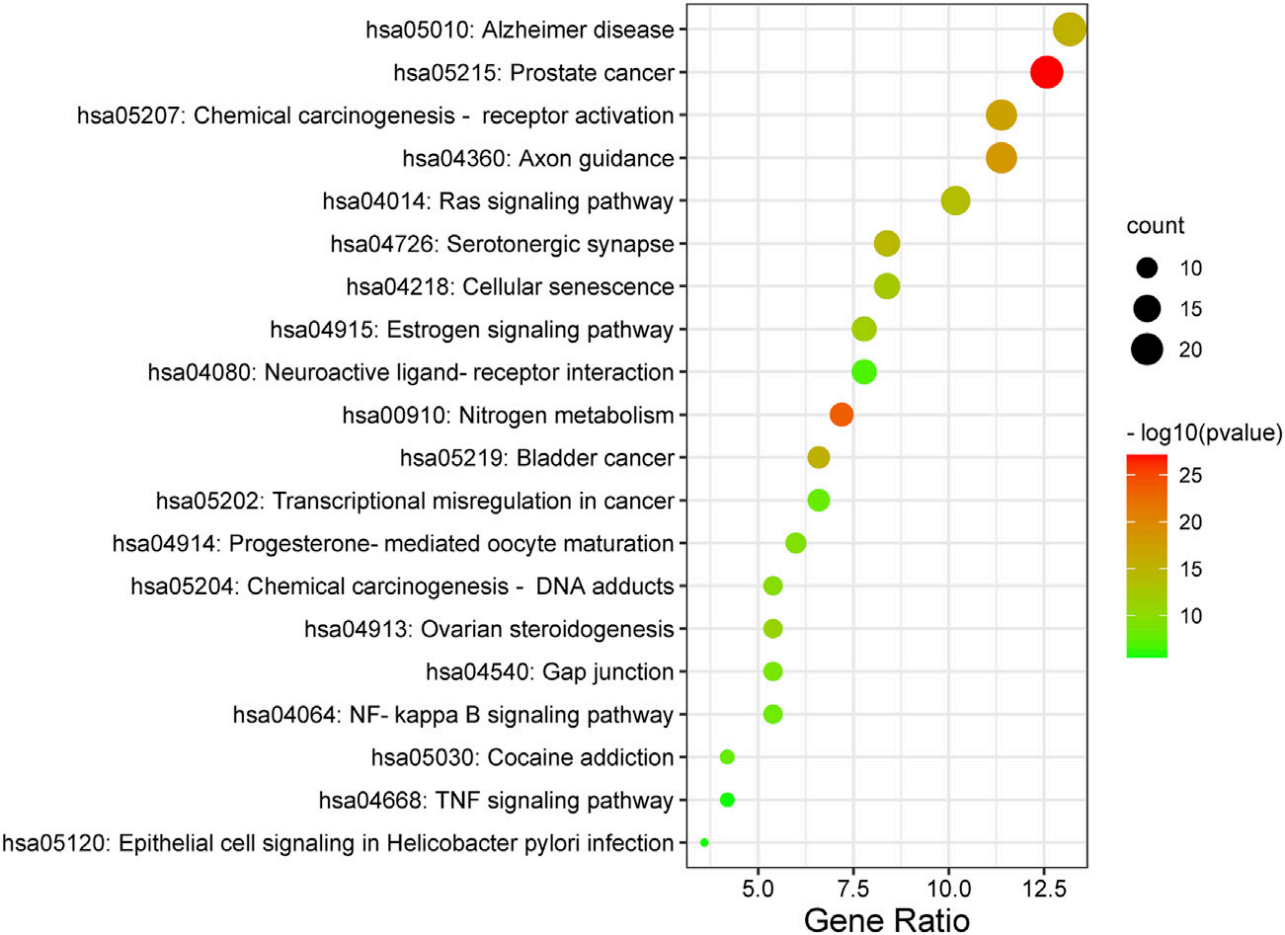
KEGG analysis of mulberroside A metabolites.

Furthermore, the Metascape was also applied in KEGG analysis. In the end, 158 signal pathways were enriched (*p* < 0.01). The result suggested that the pathways of mulberroside A metabolites were mainly involved Axon guidance, Alzheimer disease, Serotonergic synapse, NF-kappa B signaling pathway, Neuroactive ligand-receptor interaction, Epithelial cell signaling in *Helicobacter pylori* infection, TNF signaling pathway and so on (Figure 10, Supplementary Table S1).

#### 3.7.4 Construction and analysis of metabolite-target-pathway network

**FIGURE 11:**
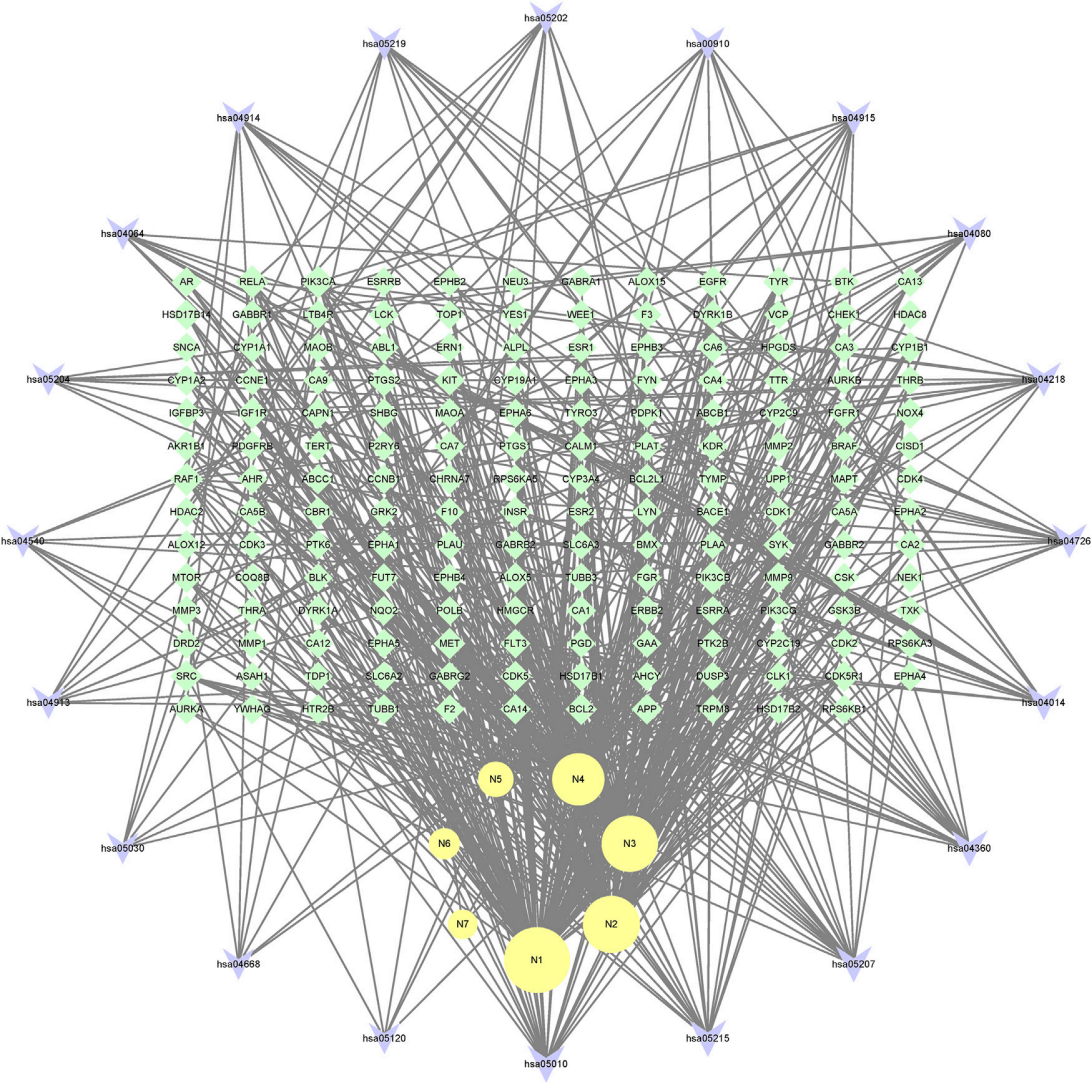
The metabolite-target-pathway network of mulberroside A metabolites.

In the end, the 7 metabolites (N1–N7), 167 targets and the top 20 pathways were imported to Cytoscape 3.8.2 for metabolite-target-pathway network. As a result, the metabolite-target-pathway network, with 194 nodes and 602 edges, was constructed, as shown in Figure 11.

Studies have shown that mulberroside A has the effect of neuroprotection (Wang C. P. et al., 2014). From the metabolite-target-pathway network, it can be seen intuitively that the metabolites N1, N2, N3 and N4 were involved in pathways of Axon guidance, Alzheimer disease and Serotonergic synapse. Axon guidance and serotonin (5-HT) play key roles in neuronal network (Negishi et al., 2005; Migliarini et al., 2013). Therefore, it was speculated that the metabolites N1, N2, N3 and N4 exert the neuroprotective effect by Axon guidance and Serotonergic synapse. Besides, oxyresveratrol (N3) has the effect of neuroprotection (Lee et al., 2019) and can be used to treat alzheimer disease (Lakshmi et al., 2021), which proved the results. From the metabolite-target-pathway network (Figure 11), it could be seen intuitively that EGFR may play an important role. EGFR is a tyrosine kinase which can regulate cellular homeostasis. Duan’s study suggested that mulberroside A could suppress the migration and invasion of renal cancer A498 cells through inhibit the expression of p-EGFR (Duan et al., 2019). So, it was indicated that mulberroside A could exert nephroprotective effect by regulating the expression of EGFR. In the pathway of Melanogenesis, tyrosinase (TYR) and tyrosinase-related protein 1 (TYRP1) are key proteins in melanin production. The study of Wang et al. showed that mulberroside A can suppress the activity of tyrosinase (Wang S. et al., 2014). Thus, it can be concluded that mulberroside A could inhibit the production of melanin by inhibiting the activity of tyrosinase.

Furthermore, metabolites N1, N2, N3 and N4 were also associated with NF-kappa B signaling pathway, TNF signaling pathway and Epithelial cell signaling in *Helicobacter pylori* infection. RPS6KA5 was the key target of N2, which exerts anti-inflammatory effect by decreasing the expression of TNF-α and inhibiting the activation of NF-κB (Wang C. P. et al., 2014). It should be noted that NF-kappa B signaling pathway plays a crucial role in mulberroside A-induced suppression of P-Glycoprotein (Li et al., 2014) and research has shown that insulin regulates P-glycoprotein via PKC/NF-κB pathway (Liu et al., 2009). Thus, it was speculated that mulberroside A has the inflammatory and antidiabetic effects after being metabolized to N1, N2, N3 and N4.

## 4 Conclusion

In the present study, an integrated strategy based on the UHPLC-Q-Exactive Plus Orbitrap MS and network pharmacology was firstly established to elucidate the metabolic mechanism of mulberroside A. In order to detect as many metabolites as possible, not only biological samples (plasma, urine, feces, liver tissues) were prepared *in vivo* of rats, but also the liver microsomes of rats were incubated *in vitro*. For the treatment of plasma samples, three methods (solid phase extraction, methanol precipitation and acetonitrile precipitation) were applied. We concluded that solid phase extraction was the most suitable method for plasma sample of mulberroside A. As a result, a total of 72 metabolites including mulberroside A were detected by UHPLC-Q-Exactive Plus Orbitrap MS. The metabolites and metabolic pathways of mulberroside A *in vivo* and *in vitro* of rats were also elaborated. The main metabolite reactions of mulberroside A were hydrolysis, glucuronidation and hydrogenation. Furthermore, the results of network pharmacology study showed that the component that exerts pharmacological effects in the body is not mulberroside A but its metabolites, which mainly include oxyresveratrol (aglycon of mulberroside A), dehydrogenation, hydrogenation, and demethylated products of oxyresveratrol.

The integrated strategy established in our study could elucidate the metabolic pathways and mechanism of mulberroside A *in vivo* and *in vitro* of rats more comprehensively, which would provide more theoretical basis for the study of mulberroside A. It would be conducive to better understand the metabolic action and promote research for the further potential therapeutic application of mulberroside A.

## Data Availability

The original contributions presented in the study are included in the article/Supplementary Material, further inquiries can be directed to the corresponding author.
