# Supplementary material for: Identification and mechanism prediction of mulberroside A metabolites in vivo and in vitro of rats using an integrated strategy of UHPLC-Q-Exactive Plus Orbitrap MS and network pharmacology
Source: Front Chem. 2022 Sep 27;10:981173. doi: 10.3389/fchem.2022.981173 (PMC9552072; doi:10.3389/fchem.2022.981173)
Supplement: Supplementary file 1 [file DataSheet1.DOCX]

Supplementary Material

# Supplementary Table

**Table S1** The KEGG enrichment results of mulberroside A.

| GO | Description | Count | % | Log10(P) |
| --- | --- | --- | --- | --- |
| hsa05215 | Prostate cancer | 21 | 12.57 | -27.13 |
| hsa05200 | Pathways in cancer | 34 | 20.36 | -25.53 |
| hsa04151 | PI3K-Akt signaling pathway | 27 | 16.17 | -22.21 |
| hsa01521 | EGFR tyrosine kinase inhibitor resistance | 16 | 9.58 | -20.24 |
| hsa01522 | Endocrine resistance | 16 | 9.58 | -18.63 |
| hsa05205 | Proteoglycans in cancer | 18 | 10.78 | -15.95 |
| hsa05224 | Breast cancer | 15 | 8.98 | -14.34 |
| hsa05226 | Gastric cancer | 15 | 8.98 | -14.25 |
| hsa05206 | MicroRNAs in cancer | 19 | 11.38 | -13.94 |
| hsa05417 | Lipid and atherosclerosis | 16 | 9.58 | -13.10 |
| hsa05230 | Central carbon metabolism in cancer | 11 | 6.59 | -12.76 |
| hsa05212 | Pancreatic cancer | 11 | 6.59 | -12.35 |
| hsa04510 | Focal adhesion | 15 | 8.98 | -12.33 |
| hsa04012 | ErbB signaling pathway | 11 | 6.59 | -11.80 |
| hsa04072 | Phospholipase D signaling pathway | 13 | 7.78 | -11.65 |
| hsa04722 | Neurotrophin signaling pathway | 12 | 7.19 | -11.51 |
| hsa05218 | Melanoma | 10 | 5.99 | -11.09 |
| hsa05225 | Hepatocellular carcinoma | 13 | 7.78 | -10.95 |
| hsa05214 | Glioma | 10 | 5.99 | -10.91 |
| hsa05167 | Kaposi sarcoma-associated herpesvirus infection | 13 | 7.78 | -10.16 |
| hsa04919 | Thyroid hormone signaling pathway | 11 | 6.59 | -10.10 |
| hsa05221 | Acute myeloid leukemia | 9 | 5.39 | -9.90 |
| hsa04664 | Fc epsilon RI signaling pathway | 9 | 5.39 | -9.84 |
| hsa05223 | Non-small cell lung cancer | 9 | 5.39 | -9.61 |
| hsa05220 | Chronic myeloid leukemia | 9 | 5.39 | -9.40 |
| hsa05163 | Human cytomegalovirus infection | 13 | 7.78 | -9.36 |
| hsa04066 | HIF-1 signaling pathway | 10 | 5.99 | -9.27 |
| hsa05165 | Human papillomavirus infection | 15 | 8.98 | -9.25 |
| hsa04062 | Chemokine signaling pathway | 12 | 7.19 | -9.07 |
| hsa05213 | Endometrial cancer | 8 | 4.79 | -8.95 |
| hsa04150 | mTOR signaling pathway | 11 | 6.59 | -8.94 |
| hsa05210 | Colorectal cancer | 9 | 5.39 | -8.91 |
| hsa05161 | Hepatitis B | 11 | 6.59 | -8.74 |
| hsa04611 | Platelet activation | 10 | 5.99 | -8.71 |
| hsa04910 | Insulin signaling pathway | 10 | 5.99 | -8.29 |
| hsa04917 | Prolactin signaling pathway | 8 | 4.79 | -8.28 |
| hsa04140 | Autophagy - animal | 10 | 5.99 | -8.17 |
| hsa04660 | T cell receptor signaling pathway | 9 | 5.39 | -8.17 |
| hsa04931 | Insulin resistance | 9 | 5.39 | -8.02 |
| hsa05131 | Shigellosis | 12 | 7.19 | -7.83 |
| hsa04662 | B cell receptor signaling pathway | 8 | 4.79 | -7.73 |
| hsa05160 | Hepatitis C | 10 | 5.99 | -7.72 |
| hsa04071 | Sphingolipid signaling pathway | 9 | 5.39 | -7.65 |
| hsa05235 | PD-L1 expression and PD-1 checkpoint pathway in cancer | 8 | 4.79 | -7.45 |
| hsa04213 | Longevity regulating pathway - multiple species | 7 | 4.19 | -7.28 |
| hsa04210 | Apoptosis | 9 | 5.39 | -7.15 |
| hsa05231 | Choline metabolism in cancer | 8 | 4.79 | -7.12 |
| hsa04625 | C-type lectin receptor signaling pathway | 8 | 4.79 | -6.92 |
| hsa04152 | AMPK signaling pathway | 8 | 4.79 | -6.44 |
| hsa04211 | Longevity regulating pathway | 7 | 4.19 | -6.18 |
| hsa04650 | Natural killer cell mediated cytotoxicity | 8 | 4.79 | -6.15 |
| hsa04370 | VEGF signaling pathway | 6 | 3.59 | -6.04 |
| hsa05135 | Yersinia infection | 8 | 4.79 | -6.00 |
| hsa04613 | Neutrophil extracellular trap formation | 9 | 5.39 | -5.92 |
| hsa04929 | GnRH secretion | 6 | 3.59 | -5.82 |
| hsa01524 | Platinum drug resistance | 6 | 3.59 | -5.49 |
| hsa04630 | JAK-STAT signaling pathway | 8 | 4.79 | -5.45 |
| hsa04810 | Regulation of actin cytoskeleton | 9 | 5.39 | -5.43 |
| hsa04024 | cAMP signaling pathway | 9 | 5.39 | -5.38 |
| hsa04380 | Osteoclast differentiation | 7 | 4.19 | -5.13 |
| hsa04923 | Regulation of lipolysis in adipocytes | 5 | 2.99 | -4.83 |
| hsa04550 | Signaling pathways regulating pluripotency of stem cells | 7 | 4.19 | -4.81 |
| hsa04666 | Fc gamma R-mediated phagocytosis | 6 | 3.59 | -4.77 |
| hsa05415 | Diabetic cardiomyopathy | 8 | 4.79 | -4.74 |
| hsa04725 | Cholinergic synapse | 6 | 3.59 | -4.39 |
| hsa05211 | Renal cell carcinoma | 5 | 2.99 | -4.39 |
| hsa04960 | Aldosterone-regulated sodium reabsorption | 4 | 2.40 | -4.29 |
| hsa04935 | Growth hormone synthesis, secretion and action | 6 | 3.59 | -4.27 |
| hsa05132 | Salmonella infection | 8 | 4.79 | -4.11 |
| hsa04930 | Type II diabetes mellitus | 4 | 2.40 | -3.91 |
| hsa04932 | Non-alcoholic fatty liver disease | 6 | 3.59 | -3.64 |
| hsa05100 | Bacterial invasion of epithelial cells | 4 | 2.40 | -3.05 |
| hsa05164 | Influenza A | 5 | 2.99 | -2.57 |
| hsa05020 | Prion disease | 6 | 3.59 | -2.37 |
| hsa05168 | Herpes simplex virus 1 infection | 8 | 4.79 | -2.19 |
| hsa00562 | Inositol phosphate metabolism | 3 | 1.80 | -2.11 |
| hsa05017 | Spinocerebellar ataxia | 4 | 2.40 | -2.08 |
| hsa00910 | Nitrogen metabolism | 12 | 7.19 | -23.48 |
| hsa04360 | Axon guidance | 19 | 11.38 | -18.25 |
| hsa05207 | Chemical carcinogenesis - receptor activation | 19 | 11.38 | -16.99 |
| hsa05208 | Chemical carcinogenesis - reactive oxygen species | 16 | 9.58 | -12.85 |
| hsa05219 | Bladder cancer | 11 | 6.59 | -15.54 |
| hsa05010 | Alzheimer disease | 22 | 13.17 | -15.48 |
| hsa05022 | Pathways of neurodegeneration - multiple diseases | 22 | 13.17 | -13.56 |
| hsa04726 | Serotonergic synapse | 14 | 8.38 | -14.50 |
| hsa00590 | Arachidonic acid metabolism | 9 | 5.39 | -10.28 |
| hsa04014 | Ras signaling pathway | 17 | 10.18 | -13.78 |
| hsa04015 | Rap1 signaling pathway | 16 | 9.58 | -13.26 |
| hsa04010 | MAPK signaling pathway | 18 | 10.78 | -13.21 |
| hsa04520 | Adherens junction | 9 | 5.39 | -9.67 |
| hsa04020 | Calcium signaling pathway | 10 | 5.99 | -6.01 |
| hsa04218 | Cellular senescence | 14 | 8.38 | -12.64 |
| hsa05203 | Viral carcinogenesis | 15 | 8.98 | -12.23 |
| hsa05170 | Human immunodeficiency virus 1 infection | 14 | 8.38 | -10.82 |
| hsa04110 | Cell cycle | 11 | 6.59 | -9.91 |
| hsa04115 | p53 signaling pathway | 9 | 5.39 | -9.56 |
| hsa05222 | Small cell lung cancer | 9 | 5.39 | -8.64 |
| hsa05169 | Epstein-Barr virus infection | 11 | 6.59 | -7.73 |
| hsa05166 | Human T-cell leukemia virus 1 infection | 11 | 6.59 | -7.31 |
| hsa05162 | Measles | 9 | 5.39 | -7.07 |
| hsa04933 | AGE-RAGE signaling pathway in diabetic complications | 8 | 4.79 | -7.05 |
| hsa04915 | Estrogen signaling pathway | 13 | 7.78 | -12.04 |
| hsa05418 | Fluid shear stress and atherosclerosis | 10 | 5.99 | -8.23 |
| hsa04926 | Relaxin signaling pathway | 9 | 5.39 | -7.35 |
| hsa04750 | Inflammatory mediator regulation of TRP channels | 7 | 4.19 | -5.90 |
| hsa04670 | Leukocyte transendothelial migration | 6 | 3.59 | -4.37 |
| hsa04913 | Ovarian steroidogenesis | 9 | 5.39 | -11.01 |
| hsa00140 | Steroid hormone biosynthesis | 7 | 4.19 | -7.33 |
| hsa05204 | Chemical carcinogenesis - DNA adducts | 9 | 5.39 | -9.78 |
| hsa00982 | Drug metabolism - cytochrome P450 | 7 | 4.19 | -6.82 |
| hsa00980 | Metabolism of xenobiotics by cytochrome P450 | 7 | 4.19 | -6.58 |
| hsa00591 | Linoleic acid metabolism | 5 | 2.99 | -6.29 |
| hsa00830 | Retinol metabolism | 4 | 2.40 | -3.25 |
| hsa04914 | Progesterone-mediated oocyte maturation | 10 | 5.99 | -9.55 |
| hsa04068 | FoxO signaling pathway | 10 | 5.99 | -8.48 |
| hsa04114 | Oocyte meiosis | 10 | 5.99 | -8.48 |
| hsa04540 | Gap junction | 9 | 5.39 | -8.82 |
| hsa05130 | Pathogenic Escherichia coli infection | 7 | 4.19 | -3.93 |
| hsa04064 | NF-kappa B signaling pathway | 9 | 5.39 | -8.17 |
| hsa05145 | Toxoplasmosis | 6 | 3.59 | -4.42 |
| hsa04137 | Mitophagy - animal | 3 | 1.80 | -2.13 |
| hsa05030 | Cocaine addiction | 7 | 4.19 | -8.01 |
| hsa05012 | Parkinson disease | 11 | 6.59 | -6.52 |
| hsa05034 | Alcoholism | 9 | 5.39 | -5.97 |
| hsa00380 | Tryptophan metabolism | 5 | 2.99 | -5.46 |
| hsa05031 | Amphetamine addiction | 5 | 2.99 | -4.39 |
| hsa04728 | Dopaminergic synapse | 6 | 3.59 | -4.02 |
| hsa00350 | Tyrosine metabolism | 3 | 1.80 | -2.98 |
| hsa05202 | Transcriptional misregulation in cancer | 11 | 6.59 | -7.96 |
| hsa04080 | Neuroactive ligand-receptor interaction | 13 | 7.78 | -7.03 |
| hsa04727 | GABAergic synapse | 6 | 3.59 | -4.98 |
| hsa05032 | Morphine addiction | 6 | 3.59 | -4.93 |
| hsa05033 | Nicotine addiction | 4 | 2.40 | -4.15 |
| hsa04723 | Retrograde endocannabinoid signaling | 4 | 2.40 | -2.03 |
| hsa05120 | Epithelial cell signaling in Helicobacter pylori infection | 6 | 3.59 | -5.59 |
| hsa04668 | TNF signaling pathway | 7 | 4.19 | -5.51 |
| hsa04657 | IL-17 signaling pathway | 6 | 3.59 | -4.85 |
| hsa05171 | Coronavirus disease - COVID-19 | 8 | 4.79 | -4.32 |
| hsa04912 | GnRH signaling pathway | 6 | 3.59 | -4.87 |
| hsa04371 | Apelin signaling pathway | 6 | 3.59 | -3.89 |
| hsa04921 | Oxytocin signaling pathway | 6 | 3.59 | -3.65 |
| hsa04916 | Melanogenesis | 5 | 2.99 | -3.60 |
| hsa05152 | Tuberculosis | 6 | 3.59 | -3.29 |
| hsa04261 | Adrenergic signaling in cardiomyocytes | 4 | 2.40 | -2.01 |
| hsa04934 | Cushing syndrome | 7 | 4.19 | -4.58 |
| hsa04610 | Complement and coagulation cascades | 5 | 2.99 | -3.95 |
| hsa04928 | Parathyroid hormone synthesis, secretion and action | 5 | 2.99 | -3.50 |
| hsa04730 | Long-term depression | 4 | 2.40 | -3.46 |
| hsa04720 | Long-term potentiation | 4 | 2.40 | -3.28 |
| hsa00790 | Folate biosynthesis | 3 | 1.80 | -3.41 |
| hsa01523 | Antifolate resistance | 3 | 1.80 | -3.18 |
| hsa05134 | Legionellosis | 3 | 1.80 | -2.41 |
| hsa04976 | Bile secretion | 4 | 2.40 | -2.82 |
| hsa04141 | Protein processing in endoplasmic reticulum | 5 | 2.99 | -2.57 |
| hsa05014 | Amyotrophic lateral sclerosis | 7 | 4.19 | -2.37 |
| hsa04659 | Th17 cell differentiation | 4 | 2.40 | -2.51 |
| hsa04340 | Hedgehog signaling pathway | 3 | 1.80 | -2.43 |
| hsa00983 | Drug metabolism - other enzymes | 3 | 1.80 | -2.00 |
